# Effectiveness of educational interventions for healthcare workers on vaccination dialogue with older adults: a systematic review

**DOI:** 10.1186/s13690-024-01260-1

**Published:** 2024-03-12

**Authors:** Manuela Dominique Wennekes, Tímea Almási, Renske Eilers, Fruzsina Mezei, Zsuzsanna Ida Petykó, Aura Timen, Zoltán Vokó, VITAL Consortium

**Affiliations:** 1 Centre for Infectious Disease Control, National Institute of Public Health and the Environment (RIVM), Bilthoven, The Netherlands; 2 Athena Institute, VU University Amsterdam, Amsterdam, The Netherlands; 3 Syreon Research Institute, Budapest, Hungary; 4 Department of Primary and Community Care, Radboud University Medical Center, Nijmegen, The Netherlands; 5 Center for Health Technology Assessment, Semmelweis University, Budapest, Hungary

**Keywords:** Healthcare worker, Older adult, Education, Intervention, Immunization, Vaccination rates, Attitude, Knowledge

## Abstract

**Background:**

Healthcare workers (HCW) significantly influence older adults’ vaccine acceptance. This systematic review aimed to identify effective educational interventions for HCWs that could enhance their ability to engage in a dialogue with older adults on vaccination.

**Methods:**

Medline, Scopus, Cochrane library and grey literature were searched for comparative studies investigating educational interventions concerning older adult vaccinations. The search encompassed all languages and publication years. Analysis was performed on the outcomes ‘vaccines offered or ordered’ and ‘vaccination rates’. Whenever feasible, a sub-analysis on publication year was conducted. Methodological limitations were assessed using the RoB 2 for RCTs and the GRADE checklist for non-randomized studies. Study outcomes were categorized according to the four-level Kirkpatrick model (1996) for effectiveness: reaction, learning, behaviour, and results.

**Results:**

In total, 48 studies met all inclusion criteria. Most studies included reminder systems signalling HCWs on patients due for vaccination. Other interventions included seminars, academic detailing and peer-comparison feedback. Four articles reporting on the reaction-level indicated that most HCWs had a favourable view of the intervention. Two of the six articles reporting on the learning-level observed positive changes in attitude or knowledge due to the intervention. Seventeen studies reported on the behaviour-level. An analysis on eleven out of seventeen studies focusing on vaccines ‘ordered’ or ‘offered’ outcomes suggested that tailored reminders, particularly those implemented before 2000, were the most effective. Out of 34 studies reporting on the result-level, 24 were eligible for analysis on the outcome ‘vaccination rate’, which showed that compared to usual care, multicomponent interventions were the most effective, followed by tailored reminders, especially those predating 2000. Nonetheless, tailored reminders often fell short compared to other interventions like standing orders or patient reminders. In both the behaviour-level and result-level ‘education only’ interventions frequently underperformed relative to other interventions. Seventeen out of the 27 RCTs, and seven of the 21 non-randomized studies presented a low-to-medium risk for bias in the studies’ findings.

**Conclusions:**

Tailored reminders and multicomponent interventions effectively assist HCWs in addressing vaccines with older adults. However, education-only interventions appear to be less effective compared to other interventions.

**Supplementary Information:**

The online version contains supplementary material available at 10.1186/s13690-024-01260-1.

**Table:** 

**Text box 1. Contributions to the literature**	
• This systematic review adds to the literature by examining the effectiveness of educational interventions with a focus on vaccination dialogue between healthcare workers and older adults.	
• In comparison to previous reviews, we adopt a broader definition of educational interventions.	
• Furthermore, we evaluate effectiveness using multiple dimensions, and we incorporate all types of older adult vaccinations.	
• Our research shows that tailored reminders were most effective in changing healthcare worker behaviour concerning vaccines. Furthermore, multicomponent interventions most effectively increased vaccination rates and healthcare worker knowledge on older adult vaccines.	

## Introduction

The proportion of older adults in the European population is rapidly increasing [1]. Older adults, defined by the WHO as those aged 60 and over [2], are more vulnerable to infectious diseases due to immunosenescence and comorbidities [3]. Vaccines against various infectious diseases like influenza, pneumococcal disease, herpes zoster, and tetanus offer protection, yet their uptake in older adults remains low [4]. For example, only 50.8% of older adults in the European Union received the influenza vaccine in 2021 [5]. A potential reason for this low vaccine uptake is a lack of awareness among older adults about which vaccines are available to them [6].

Research underscores the pivotal role healthcare workers (HCW) play in facilitating vaccine acceptance among older adults [7–9]. Previously, we showed that older adults prefer to be informed by HCW on available vaccines [6]. It is observed that a significant factor influencing vaccine acceptance is the recommendation by an HCW. Similarly, the absence of such recommendation from a medical professional often leads to the decision not to receive a specific vaccine [8, 9].

The level of knowledge on vaccines for older adults may influence vaccine recommending behaviours among HCWs. It was found that HCWs who possess a thorough understanding of vaccines are more likely to advocate for their use among patients [10]. Educational interventions can enhance this knowledge by boosting it and improving skills [11]. HCWs themselves also recognize their need for more knowledge on older adult vaccines, expressing a need for education [12].

To the best of our knowledge, only three systematic reviews explored the effect of educational interventions aimed at HCWs, with a specific or partial focus on vaccination of older adult patients [13–15]. These three reviews showed mixed effects and have several limitations, including a rather narrow spectrum of educational interventions [13, 14], a limited range of vaccine types, and focused predominantly on ‘vaccination coverage’ or ‘increased vaccination rate’ as their primary outcome [13–15]. However, effectiveness of educational interventions can be evaluated across multiple dimensions, therefore, we conducted a systematic literature review using Kirkpatrick’s evaluation model [16].

In light of previously mentioned reviews’ limitations and in an effort to broaden the existing knowledge of HCW education’s effectiveness on older adult vaccination beyond just the result level, we conducted a systematic literature review incorporating all types of adult vaccines. Our definition of educational interventions included any intervention with a learning component such as knowledge transfer (e.g., courses, workshops, educational outreach, and feedback systems), and reinforcement (e.g., reminder systems). Despite previous reviews often not considering reminder systems as an educational intervention [13–15], this study viewed reminders as a form of learning through repetition [17]. The theory is that repetitive exposure to reminders would engrain the information deeper into the minds of HCWs, reminding them to offer vaccination.

The primary objective of this study was to identify the characteristics of effective educational interventions designed for HCWs that enhance their ability to engage in a dialogue with older adults on vaccination.

## Methods

### Search strategy

We systematically searched academic databases, specifically MEDLINE (via PubMed) and Scopus on March 31, 2020 and the Cochrane library on May 19, 2020. Our search strategy incorporated a combination of search strings to encompass all relevant keywords and their synonyms that might appear in papers to describe HCWs (e.g., healthcare, general practitioner, GP, nurse, doctor), educational interventions (e.g., educate, teach, train, learn, academic detailing, remind*), vaccines (e.g., inoculat*, vacc*, immunization), adult population (e.g., “older adult”, elderly, “old age”, senior). Certain terms (e.g., cross-sectional studies, matern*, parent*, pediatric*, poliomyelitis, mumps, tuberculosis) mostly pertaining to vaccines for children or pregnant women, were excluded. The cross-sectional study design was excluded as it cannot be used to study causality. The complete search strategies can be found in appendix 1–3. In addition to these academic resources, we also searched grey literature sources in May and June 2020. An overview of the searched grey literature sources is available in appendix 4. We further augmented our search through articles received from professional contacts. All identified titles and abstracts were imported into Covidence (Veritas Health Innovation). To avoid redundancy, the system initially de-duplicated the search results. Any duplicates not recognized by the system were manually removed, tagged as ‘duplicate’. Independent reviewers (MW, TA, RE and ZP) performed both the title/abstract and full-text screening twice to ensure thoroughness.

Disagreements among reviewers were reconciled through discussion. All records that were included during the title-abstract screening underwent full-text examination. After completing the full-text review, MW and TA manually combed through the reference lists of the included research papers and reviews to identify any additional pertinent articles. Reviews discovered during this manual search were also scrutinized for relevant studies. Finally, after completing data extraction, the searches were re-executed in the academic databases on November 6, 2020.

### Inclusion and exclusion criteria

We included studies regardless of language, geographical location and study year, provided that the interventions used remained relevant in the contemporary society. To evaluate the efficacy of interventions, we only included comparative studies. Anticipating dearth of randomized controlled trials (RCTs) in our research topic, we also included non-randomized controlled trials, controlled before-after studies and observational studies, with the exception of cross-sectional studies. Our primary population of interest was HCWs, encompassing individuals with a (para)medical training who provide (health)care to clients. This includes HCWs in primary and secondary care, as well as social workers. Our secondary population of interest consisted of older adults. In addition to the age group as defined by the WHO, we also included those aged 50–59 years as we were interested in this category that precedes the indicated age-group for vaccination. However, we also included articles focusing on older adults and groups at high-risk for complications as a result of infectious diseases simultaneously, given their common occurrence in the literature. Therefore, we included articles that exclusively focused on older adults and articles with patient groups comprised of both older adults and younger high-risk patients. Studies examining at least one educational intervention were deemed eligible, and we placed no limit on the number of interventions compared within a single study. We did not impose any specific exclusion criterion on eligible comparators.

**Fig. 1:**
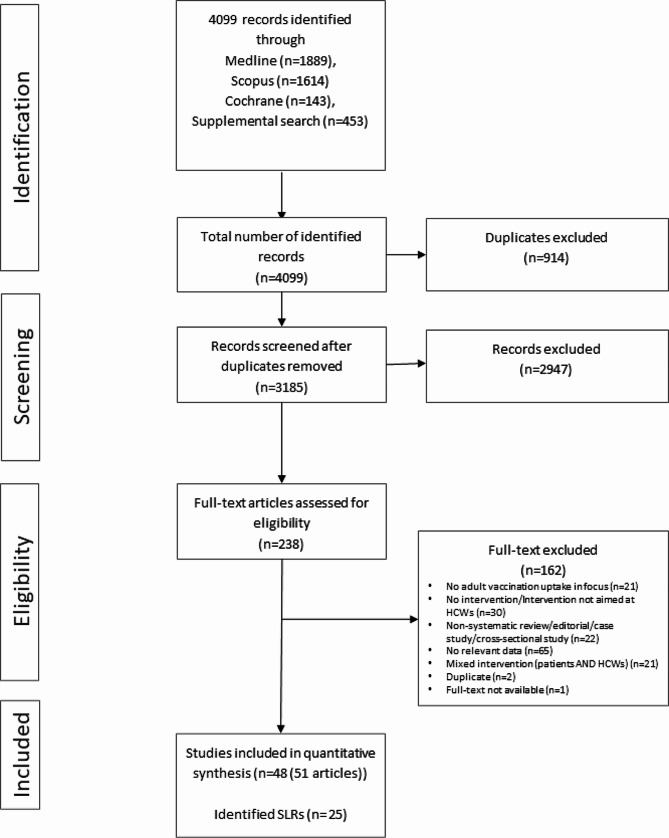
Study selection

Our exclusion criteria were constructed hierarchically; thus if a record was not excluded based on the first criterion, it was then evaluated against the subsequent criteria. Figure 1 provides an overview of the exclusion criteria. We excluded articles featuring ‘mixed interventions’ that simultaneously targeted HCWs and older adults, as it would be impossible to discern the source of the results. Lastly, we excluded informal caregivers (e.g., relatives) from the primary population, and from the secondary population, we excluded children and young adults, unless they were part of mixed patient groups that also comprised adults.

### Data extraction

Four independent reviewers (MW, TA, RE and FM) extracted data from the included studies into a designated data extraction table (MS Excel). A random study was selected for pilot extraction. Following the pilot extraction, the data extraction grid was finalized based on reviewers’ feedback. It was subsequently circulated to all reviewers, accompanied by an example of a study extraction for guidance. The data extraction process unfolded in three stages. Initially, one reviewer extracted data from each article. Next, a different reviewer cross-checked the extracted data. Finally, any disagreements were resolved through discussion until a consensus was reached. A comprehensive list of extracted data items can be found in Appendix 5. Data on the general study characteristics (e.g., country/geographical location, healthcare setting and research question), author’s contact details, methods (e.g., design, pre-specified outcomes), intervention (e.g., content of intervention, didactical methods, satisfaction with the intervention, missing data), primary and secondary population (e.g., inclusion and exclusion criteria, group differences at baseline, number of participants included), outcomes (e.g., outcome type, before and after data), and the patient pool used to calculate the results (e.g., all patients registered, eligible patients only) were extracted.

### Assessment of bias

We assessed the quality of included articles using the Revised Cochrane risk-of-bias tool (RoB 2) [18] for RCTs. The Grading of Recommendations Assessment, Development and Evaluation (GRADE) checklist was used to assess the risk of bias in non-randomized study [19] (Appendix 6). Risk of bias assessment was conducted by outcome level by one reviewer, and cross-checked by a second reviewer. Any disagreements were resolved through discussion until consensus was reached. The associated risk for each article is presented in Table 2, and 3; Figs. 4 and 5.

### Data analysis

The Kirkpatrick evaluation model, a commonly used framework, appraises effectiveness at four levels: reaction, learning, behaviour, and results [16]. This multi-level approach allows for a more nuanced understanding of why interventions may or may not be effective. The primary outcomes of the included studies were categorized according to these four levels. In the context of our research topic, ‘reaction’ measures the HCW satisfaction with the delivered intervention. ‘Learning’ the extent to which the intervention enhanced HCWs’ knowledge about adult vaccines or fostered a positive attitude towards vaccinating older adults. ‘Behaviour’ captures behavioural shifts due to the intervention (e.g., increase in offering vaccines) and ‘results’ evaluate the overall outcomes of the training, such as increased vaccination rates. Secondary outcomes were patient-oriented, such as changes in patient satisfaction, reactions and behaviours, as well as decreased disease incidence.

Substantial heterogeneity in outcomes reflecting achieved changes in HCWs reaction, learning and behaviour was anticipated. Thus, we did not plan to conduct a meta-analysis. However, when sufficient data on a particular outcome, an analysis was conducted by intervention type. Moreover, when sufficient studies investigated the same combination of intervention and comparison type, published before the year 2000 and after, a subgroup-analysis based on publication year was conducted. We used the differences in respective outcomes from the baseline between the study groups as effect measure. If changes were not reported and could not be derived from the published data, we then compared the post-intervention outcome values.

This review was registered with PROSPERO (International Prospective Register of Systematic Reviews) under registration number: CRD42020180165.

## Results

### Included studies

In total, 3646 records were identified through systematic literature searches and an additional 453 records via grey literature, manual search of the reference lists of the included research articles and reviews, and articles received through professional contacts. This added up to a total of 4099 records which after duplicate removal yielded 3185 unique studies for screening. Out of these, 48 studies (described in 51 articles) were included in this review (Fig. 1).

### Characteristics of studies included

Almost half of the included studies (*n* = 22) [20–43] were published before 2000, 16 between 2000 and 2009 [44–59] and 10 from 2010 to 2020 [60–69] (Table 1). The majority of studies were conducted in the US (*n* = 37) [20–26, 28–34, 37, 39–42, 44–46, 49, 52, 53, 55–68]. Most interventions focused on physicians, among whom also residents and interns [20–23, 25–28, 30–34, 37–45, 47–50, 52, 53, 58, 60, 61, 63–66]. Some studies targeted both physicians and nurses [24, 35, 36, 57, 59, 70]. Nurses would offer vaccination to patients through standing order procedures (SOP) enabling healthcare personnel other than physicians to evaluate immunization status and administer vaccines [24, 46, 57, 59]. In some cases, both physicians and nurses received reminders [57, 70]. In one study nurses called patients to remind them they were due for vaccination [35, 36]. The study of MacIntyre, Kainer and Brown [51] engaged both primary care physicians and hospital staff in their intervention. Furthermore, one study aimed their intervention on long-term care facilities [56], another on pharmacist-technicians [62] and one on community HCW [69]. There were also some studies where it was not entirely clear what HCW-types were part of the intervention [29, 51, 54, 67, 68], referring for example to hospital staff or primary care clinics. The most frequently studied educational interventions were tailored reminders for vaccination [21, 25, 29, 31, 32, 34–39, 41, 42, 45, 46, 51, 60, 61, 63, 70]. These were tested either in the form of automatic systems (e.g., pop-up messages when opening eligible patients’ charts, etc.) or required active assistance from administrators/nurses to remind practicing physicians to vaccinate. The most targeted immunization was influenza vaccination (*n* = 33) [20–32, 36, 38, 39, 41–46, 48–51, 54, 57, 59, 60, 63, 64, 67, 69, 70], pneumococcal (*n* = 27) [22, 23, 25, 28, 30, 31, 34, 37, 39, 40, 42, 45, 46, 51–56, 58, 59, 61–65, 67] and tetanus (*n* = 8) [23, 25, 28, 33, 35, 36, 39, 47, 68], while herpes zoster was least targeted (*n* = 2) [62, 64]. Most interventions were tested in outpatient care, including primary care and family medicine (*n* = 26), while eleven studies were hospital-based.

Within the categorization of included studies according to the levels of the Kirkpatrick model, the studies were further categorized based on intervention type and applied comparison contrast, resulting in six categories: ‘general reminders (e.g., general factsheets) versus usual care’, ‘tailored reminders versus usual care’, ‘tailored reminders versus non-education intervention’, ‘education only versus other interventions’, ‘multicomponent intervention versus usual care’ and ‘other’. A ‘multicomponent’ intervention combined two or more strategies.

**Fig. 2:**
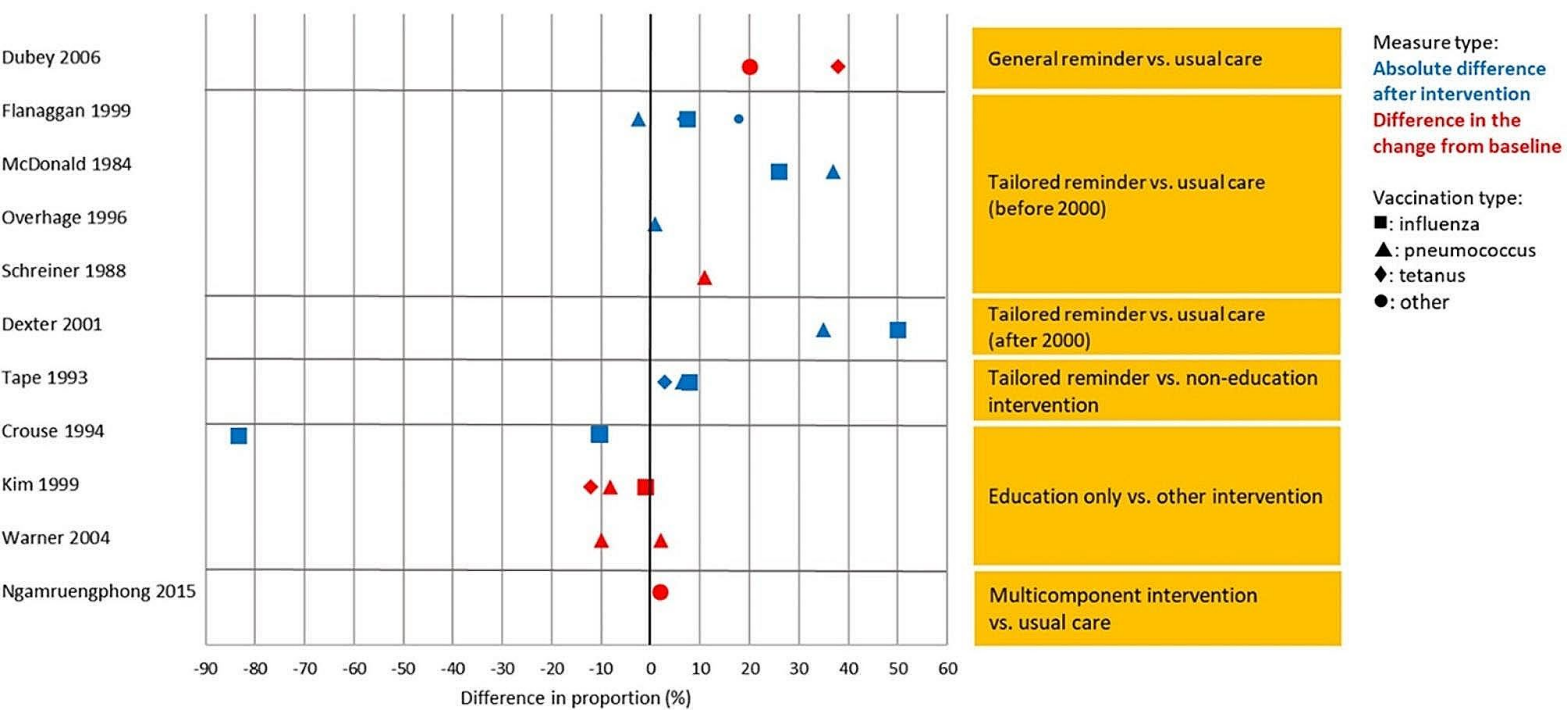
Proportion of ordered or offered vaccinations across studies by intervention contrast, outcome measure, and vaccination type

**Fig. 3:**
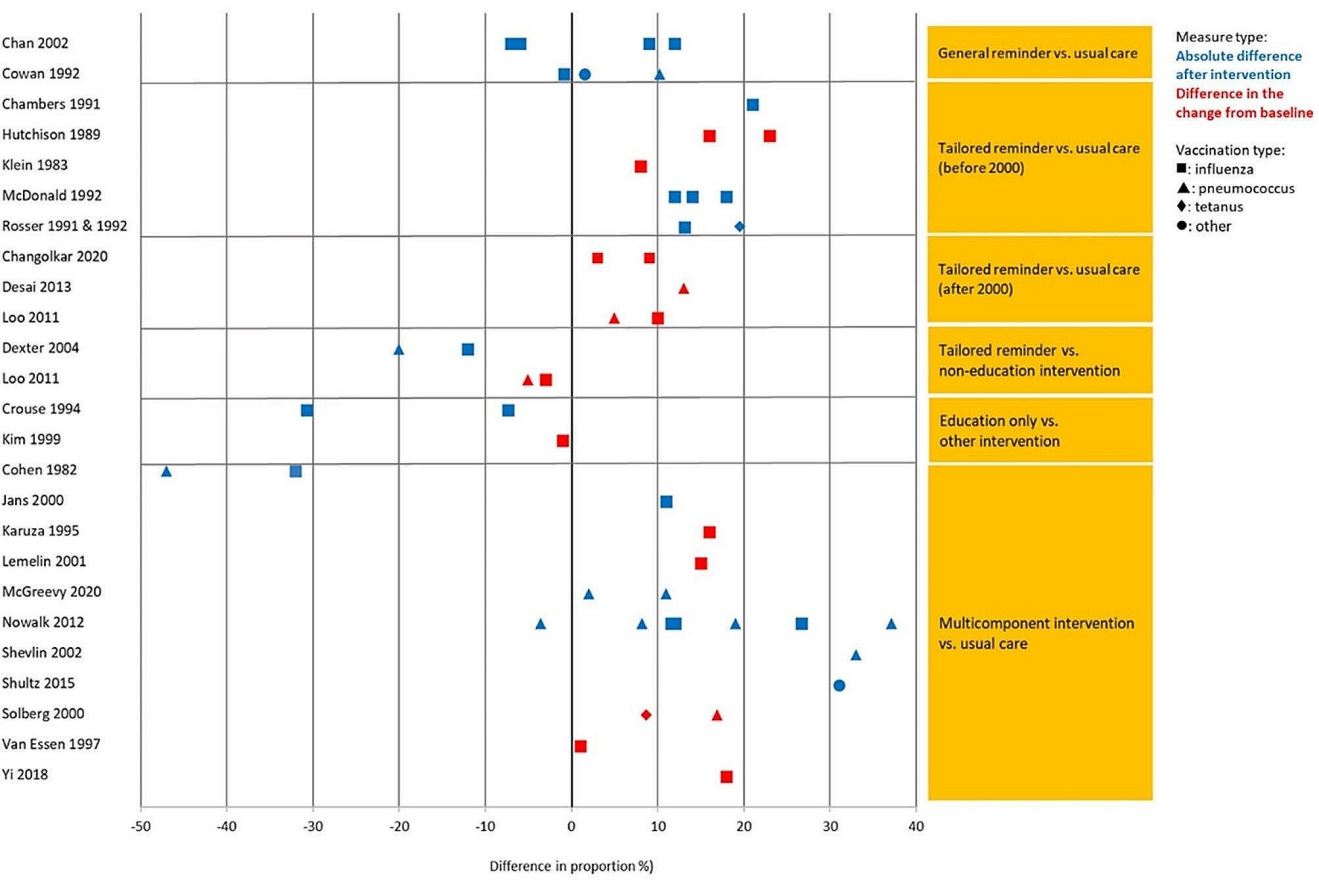
Vaccination rate across studies by intervention contrast, outcome measure, and vaccination type

Physician-dependent actions (e.g., ‘documenting vaccination status’, ‘offering vaccines’ and ‘vaccine ordering’ (for uniformity purposes regardless of whether patient consent was considered)) were categorized under level 3 ‘behaviour’, while outcomes influenced by patient’s decision on vaccine acceptance (e.g., ‘vaccination coverage’ and ‘vaccination rates’), were categorized under level 4 ‘results’. Certain studies included more than one outcome type. In such cases the paper may appear in multiple sub-sections. Due to data scarcity and different measurement tools, only “vaccination rates” and “ordered or offered vaccines” (Kirkpatrick [16] level 3 ‘behaviour’ and 4 ‘results’) outcomes allowed for comparative analysis by intervention type and comparison contrast (Figs. 2 and 3). In-depth discussions were limited to studies with significant results. Nevertheless, an overview of the characteristics of all studies included in this systematic review can be found in Table 1 (see appendix 7 for additional study details).

**Table 1:** Characteristics of the included studies

	HCPs		Patients				
Study	Intervention	Control	Intervention	Control	Targeted population	Intervention type(s)	Main outcome(s)
Calkins, Katz [20]	17 primary care providers (5 practices)	19 primary care physicians (4 practices)	45	45	65+	Small-group consensus process	Vaccination rates
Chambers, Balaban [21]	Physicians (*n* = 32) were stratified by level of training and randomized to one of three groups,	See under ‘Intervention’	-Always reminded: 271 -Sometimes reminded: 146	-Never reminded: 218	65 + or risk groups	Reminder (digital)	Vaccination rates
Chan, MacLehose and Houck [44]	-Solo practices 1997: 23 -Group practices 1997: 28 -Solo practices 1998: 20 -Group practices 1998: 32	-Solo practices 1997: 21 -Group practices 1997: 33 -Solo practices 1998: 20 -Group practices 1998: 27	-Solo practices 1997: 1486 -Group practices 1997: 1341 -Solo practices 1998: 561 -Group practices 1998: 868	-Solo practices 1997: 596 -Group practices 1997: 877 -Solo practices 1998: 1310 -Group practices 1998: 1286	Patients with a chronic medical condition	Reminder (paper)	Vaccination rates
Changolkar, Rewley [60]	Low clin. workload: 12 physicians High clin. workload: 18 physicians	Low clin. workload: 3 physicians High clin. workload: 23 physicians	Low clin. workload: before:1974 (pre-intervention), 1799 (post) High clin. workload: 6397 (pre-intervention), 5342 (post)	Low clin. workload:558 (pre-intervention), 516 (post) High clin. workload:14,275 (pre-intervention), 14,549 (post)	Mean age (SD): 58.7 (16.3) years	Reminder	Vaccination rates
Cohen, Littenberg [22]	22 physicians (2 firms)	1 firm (no info on number of physicians)	Eligible patients -Pneumovax: 547 -Influenza: 581	Eligible patients -Pneumovax: 291 -Influenza: 291	65+	Education + reminder (checklist, paper)	-Vaccination rates -Knowledge
Cowan, Heckerling and Parker [23]	16 residents	13 residents	62	45	> 65 influenza and pneumococcal. Tdap every ten years	Reminder (recommend-dations, paper)	-Vaccination coverage -Knowledge -Attitude
Crouse, Nichol [24]	6 hospitals in total; 2 hospitals per intervention type	n/a	-Standing order group: 609 -Physician reminder group: 1925 Physician education group: 11,800	n/a	High-risk patients	Comparison of: -standing orders -physician education -Physician reminder (paper)	-Proportion of offered vaccination -Vaccination rates
Desai, Lu [61]	14 rheumatologists	21 rheumatologists	3267	450	-65 years and older - <64 when in immunocompromising condition/immunosuppressive therapy	Reminder (paper)	Vaccination rates
Dexter, Perkins [45]	-96 (47.5%) physicians intervention group only -28 (13.9%) physicians assigned alternately to both intervention and control group	- 78 (38.6%) physicians control group only	4995 hospitalizations	5070 hospitalizations	NI	Reminder (digital)	Proportion of offered vaccination
Dexter, Perkins [46]	4 teams assigned to physician reminder group	4 teams assigned to standing order group	691 Patients eligible for vaccination	623 Patients eligible for vaccination	− 65 years and older or risk group (relevant chronic disease)	Comparison of: - Standing orders (digital) -Physician Reminders (digital)	Vaccination rates
Dubey, Mathew [47]	20 primary care physicians	18 primary care physicians	248	261	Patients aged 21 years and older	Reminder (paper)	Proportion of offered vaccination
Flanagan, Doebbeling [25]	70 physicians	47 physicians	NI	NI	-Influenza: hospital employee or older than 64.5 years of age -Pneumococcal: recommended if age was greater than 64 years (flagged “consider” if more than 63.5 years) and more than 10 years since last received (flagged “consider” if more than 7 years since last received). -Td: recommended if no history of Tetanus vaccine in over 9 years and 6 months.	Reminder (digital)	Proportion of vaccines ordered
Hohmann, Hastings [62]	30 community pharmacies	32 control community pharmacies	NI	NI	Unclear	Comparison of: -Multicomponent intervention -Online webinar	-Vaccination coverage -Organization level
Hutchison [70]	NI	NI	Eligible patients: 593	Eligible patients: 618	65 years and older	Reminder (paper)	-Vaccination rates -Patient refusal rate
Jans, Schellevis [48]	14 practices (total 16 physicians)	5 practices (totalling 5 physicians)	455	152	16–70 years of age and having received a diagnosis of asthma or COPD	Multicomponent intervention: quality system	Vaccination rates
Karuza, Calkins [26]	23 primary care physicians	28 primary care physicians	Mean number of charts reviewed per physician: 30	Mean number of charts reviewed per physician: 29	65 years and older	Small-group consensus process	-Vaccination rates -Attitude -Knowledge
Kerse, Flicker [27]	21 general practitioners	21 general practitioners	121	112	65 years and older	Multicomponent intervention: -Clinical practice audit with feedback -Educational detailing -Card based prompt system -Seminar or home based learning -Resource directory	-Vaccination rates
Kiefe, Allison [49]	35 physicians	35 physicians	965	966	65 years and older with diabetes mellitus	Comparison of: -multi-component intervention with performance feedback -Same multicomponent intervention + achievable benchmark feedback	-Vaccination rates
Kim, Kristopaitis [28]	Multicomponent intervention: 21 primary care physicians	Education only: 20 primary care physicians	Multicomponent intervention: 905	Education only: 905	Patients aged 65 to 75 years	Comparison of: -Education only -Multicomponent intervention	-Proportion of vaccines offered -Vaccination rates
Klein and Adachi [29]	NI	NI	100	100	-Patients of 65 years or older -Patients with high-risk conditions	Reminder (paper)	Vaccination rates
Korn, Schlossberg and Rich [30]	15 residents	13 residents	-SPR site post-intervention: 199 -VA site: 149	-SPR site pre-intervention: 202 -VA site: 151	65 years and older	Multicomponent intervention	Proportion of vaccines ordered
Lemelin, Hogg and Baskerville [50]	22 practices	23 practices	NI	NI	65 years and older	Multicomponent intervention	Vaccination rates
Loo, Davis [62]	-Reminder: 17 primary care physicians -Reminder + panel manager: 17 primary care physicians	Control: 20 primary care physicians	-Reminder: 1336 -Reminder + panel manager: 1394	Control: 1930	65 years and older	Comparison of -Reminder (digital) -Reminder (digital) + panel manager -Control	Vaccination rates
Loskutova, Smail [64]	23 primary care physicians	20 primary care physicians	-Influenza year 1 (before): 20,952 -Influenza year 2 (after): 24,506 -Pneumococcal year 1 (before): 18,244 -Pneumococcal year 2 (after): 27,415 -Zoster year 2 (before): 27,415 -Zoster year 3 (after): 30,844	-Influenza year 1 (before): 15,076 -Influenza year 2 (after): 17,256 -Pneumococcal year 1 (before): 12,577 -Pneumococcal year 2 (after): 21,465 -Zoster year 2 (before): 21,465 -Zoster year 3 (after): 24,906	Influenza: 18 years and older Pneumococcal: -65 years and older -19-64 years when at least one risk factor Herpes zoster: 60 years and older	Comparison of: -Multicomponent intervention - Reminders + Clinical decision support system (algorithms for provider reminders and standing orders)	Vaccination rates
MacIntyre, Kainer and Brown [51]	NI	NI	Hospital reminder: 70 GP reminder: 61	n/a	65 years and older	Comparison of: -Hospital reminder (paper + verbal) -GP reminder (letter)	Vaccination rates
McDonald, Hui [31]	61 residents	54 residents	NI	NI	Influenza: according to U.S. Public health service criteria Pneumococcal: Over 65 years, otherwise according to U.S. Public health service criteria	Reminder (paper)	-Intentions among HCWs -Proportion of vaccines offered
McDonald, Hui and Tierney [32]	NI	NI	-Intervention group 1978–1979: 1328 -Intervention group 1979–1980: 1489 -Intervention group 1980–1981: 1316	-Control group 1978–1979: 1290 -Control group 1979–1980: 1412 -Control group 1980–1981: 1236	Older than 65 years or risk group (chronic lung disease, asthma, diabetes mellitus, congestive heart failure, severe renal/hepatic failure)	Reminder (digital)	-Vaccination coverage -Disease incidence
McGreevy, McGowan [65]	2 attending physicians and 8 residents	Not explicitly stated	≥ 65 years: 95 ≤ 65 years: 255	≥ 65 years: 103 ≤ 65 years: 242	65 years and older	Multicomponent	Vaccination rates
Ngamruengphong, Horsley-Silva [66]	20 primary care residents	19 primary care residents	29	17	Patients with diabetes mellitus	Comparison of: -Standard education + extra 30-minute lecture + pocket card + monthly e-mail reminders with the lecture content -Standard education	Knowledge
Nowalk, Nutini [67]	10 providers and 7 clinical assistants	2 providers and 3 clinical assistants	7794	2199	Influenza: all adults Pneumococcal: 65 years and older or high-risk patients aged 18–64.	Multicomponent: Toolkit	Vaccination coverage
Ornstein, Garr [33]	-Physician reminders: 14 -Patient reminders: 12 -Physician and patient reminders: 13	Control: 10	-Physician reminders: 1988 -Patient reminders: 1925 -Physician and patient reminders: 1908	Control: 1576	18 years or older	Comparison of: -Educational + administrative interventions -abovementioned interventions + reminders (paper)	Vaccination coverage
Overhage, Tierney and McDonald [34]	12 teams of physicians and medical students	12 teams of physicians and medical students	821	801	Older than 65 years or in risk group (reactive airway disease, congestive heart failure, diabetes, splenectomy, sickle cell disease)	Reminder (digital and paper)	Compliance with preventive care guidelines
Quinley and Shih [52]	-African-American practices: 118 -High volume practices: 582	-African-American practices: 100 -High volume practices: 150	NI	NI	65 years and older	Comparison of: -Mailer only -Mailer + telephone follow-up	Vaccination rates
Rosser, McDowell and Newell [36]	6 teams consisting of a staff physician, nurse, and three to five residents. No information was provided on the distribution of these HCPs per study group.	NI	-Physician reminder: 1471 -Letter reminder: 1541 -Telephone reminder	-Nonrandomized control: 2619 -Randomized control: 1403	Influenza: Older than 65 years Tetanus: Over 18 years of age	Comparison of: -Physician reminder (paper) -Letter reminder to patients -Telephone reminder to patients	Vaccination rates
Rosser, Hutchison [35]	4 teaching medical practices participated, but no information was provided on the distribution of these HCPs per study group.	NI	-Physician reminder: 1399 -Letter reminder: 1471 -Telephone reminder: 1390	Control: 1329	Patients 20 years of age or more	Comparison of: -Physician reminder (paper) -Letter reminder to patients -Telephone reminder to patients	Vaccination rates
Schreiner, Petrusa [37]	20 residents	22 residents	1260	504	-65 years and older or when having a chronic disease	-Five months with reminders (paper) followed by six months follow-up without reminders	Proportion of vaccines offered
Shevlin, Summers-Bean [53]	2 floors	2 floors	296	238	-65 years and older or when in risk group (diabetes, alcohol abuse, lung/heart disease, HIV, chronic renal failure)	-Reminders (paper) + in-service education + feedback	Vaccination rates
Shultz, Malouin [68]	5 family medicine clinics	4 primary care clinics	39,882	28,032	11- to 64-year-old patients	Intervention consisting of: -Reminder (digital) -Pay-for-performance -Monthly status reports	Vaccination rates
Siriwardena [54]	15	15	Influenza vaccination 65+: 13,633	Influenza vaccination 65+: 13,947	-65 years and older or when in risk group (coronary heart disease, diabetes, or splenectomy)	Comparison of: -Educational outreach based on principles of academic detailing + audit feedback and written guidance -Audit feedback and written guidance	Vaccination rates
Solberg, Kottke [55]	22 clinics	22 clinics	3379	3451	Older than 64 years	- Leadership involvement, training, network and consultation	Vaccination rates
Stevenson, McMahon [56]	Alaska: 26 facilities Idaho: 23 facilities Montana: 78 facilities Wyoming: 6 facilities	n/a	Alaska: 1099 Idaho: 1274 Montana: 5671 Wyoming: 882	n/a	All residents	Comparison of 4 slightly different multicomponent interventions	Vaccination rates
Tang, LaRosa [38]	13	10	NI	NI	Patients 65 years of age and older	Reminder (digital)	Behaviour among HCWs
Tape and Campbell [39]	45 residents in total and 4 attending physicians. No information on the numbers assigned to the control/intervention arm	See under ‘Intervention’	Tetanus: 937 Influenza: 212 Pneumococcal: 310	Tetanus: 870 Influenza: 172 Pneumococcal: 274	-Influenza: 65 years and older or when in risk group (diabetes, chronic respiratory or heart disease) -Pneumococcal: 65 years and older or when in risk group (immunocompromised, diabetes, chronic respiratory or heart disease) -Tetanus: all patients	Comparison of: -Education + flow sheet -Education + reminder (six months on paper, then six months displayed on terminals)	Behaviour among HCWs
Tierney, Hui and McDonald [40]	-Group A feedback and A reminders: 33 - Group A feedback and B reminders: 31 -Group B feedback and A reminders: 36	Group B feedback and B reminders: 35	-Group A feedback and A reminders: 1487 - Group A feedback and B reminders: 1451 -Group B feedback and A reminders: 1606	Group B feedback and B reminders: 1501	Unclear	Various combinations feedback and reminders (paper)	Behaviour among HCWs
Trick, Das [57]	NI	NI	-Nursing reminder: 69 -Opt-out: 66	Control:69	All patients (median age: 52 years)	Comparison of: -Standing-orders policy -Augmentation of the standing-orders policy with electronic opt-out orders for physicians -Augmentation of the standing-orders policy with electronic reminders to nurses	Vaccination rates
Turner, Waivers and O’Brien [42]	12 residents	12 residents	117	246	Both vaccines: older than 65 or in risk group	Comparison of: -Physician reminder (paper) -Physician reminder (paper) + reminder card carried by patients	Vaccination coverage
Turner, Peden and O’Brien [41]	-Computer-generated reminder: 15 physicians -Patient-carried reminder: 22 physicians	n/a	-Computer-generated reminder: 300 -Patient-carried reminder: 440	n/a	65 years or older or suffering from a chronic disease	Comparison of: -Physician reminder (paper) -Reminder card carried by patients	Vaccination coverage
van Essen, Kuyvenhoven and de Melker [43]	64 practices (84 GPs)	74 practices (88 GPs)	about 250,000	about 300,000	All patients	Guideline for influenza vaccination	-Vaccination coverage -Organization level
Warner and Seleznick [58]	1 clinic	1 clinic	NI	NI	Patients 65 years of age or older	Comparison of: -Education + reminder (paper) -Education only	-Vaccination rates -Organization level
Winston, Lindley and Wortley [59]	-A: 93 (50-64y, 2002) 90 (50-64y, 2003) 93 (65 + y, 2002) 87 (65 + y, 2003) -B: 39 (50-64y, 2002) 49 (50-64y, 2003), 98 (65 + y, 2002) 114 (65 + y, 2003) -C: 109 (50-64y, 2002), 95 (50-64y, 2003), 110 (65 + y, 2002), 96 (65 + y, 2003) -D 112 (50-64y, 2002) 91 (50-64y, 2003) 102 (65 + y, 2002) 98 (65 + y, 2003)	n/a	4 Hospitals (A-D)	n/a	Patients aged 50 years and older	Comparison of: -A. Nurse-administered standing orders protocol + Training and information: Small group meetings on individual units -B. Physician reminder program + Training and information: E-mail, newsletters, and division meetings -C + D Nurse-administered standing orders protocol + Training and information: Nurse managers trained during regular staff meetings	Vaccination rates
Yi, Zhou [69]	NI	NI	7013	5500	60 years and older + chronic disease	Education	Vaccination coverage

NI = ‘No information available’, n/a = not applicable

### Kirkpatrick level 1: reaction

Four studies reported HCW satisfaction with the intervention [24, 55, 65, 67]. One study in the categories ‘education only versus other interventions’ and ‘other’ reported on HCW satisfaction with the intervention. The study surveyed hospital staff about the implementation of an influenza vaccination program for patients. It was well received by the HCW participating [24]. Three studies in the ‘multicomponent intervention versus no intervention’ category reported on HCW satisfaction with the intervention [55, 65, 67].

McGreevy, McGowan [65] studied an intervention that involved a combination of education, nursing staff placing pending vaccination orders, and the use of pocket cards bearing recommendations. The nursing personnel reported that the implementation of this project was time-efficient, and they responded positively to the notion of a standardized vaccine protocol. This protocol would involve them reviewing medical charts prior to patient visits and attaching orders for necessary vaccines. Most of the 24 residents and attending physicians found the intervention to be between somewhat and very beneficial. Furthermore, 92% expressed strong preference for this model where nurses review medical charts and append orders for the pneumococcal vaccine.

In Solberg, Kottke [55] the intervention consisted of a multifaceted approach combining leadership support, training, consultation and networking. A notable 94% of the 114 HCWs who responded to the questionnaire indicated their satisfaction with the intervention from ‘satisfied’ to ‘very satisfied’. In addition, 91% of the respondents felt that the intervention was a worthy investment of their time.

The study conducted by Nowalk, Nutini [67] introduced a toolkit to promote the usage of SOP. From the intervention group, 67% of the HCWs spread across three practices, completed the survey after the intervention. Among these practices, one exhibited lower enthusiasm levels, which was consistent with its vaccination rates being less than the two other practices (see level 4, vaccination rate). However, the other two practices reported unanimous intention to continue using the toolkit for both influenza and pneumococcal vaccines. In contrast, this was 88.9% and 77.8% for each vaccine, respectively in the less enthusiastic practice.

### Kirkpatrick level 2: learning

Six studies reported on knowledge or attitude [22, 23, 26, 34, 41, 66] with two showing significant benefit in the intervention category ‘multicomponent intervention versus usual care’ [22, 66].

#### Multicomponent intervention versus usual care

Cohen, Littenberg [22] assessed changes in attitudes and knowledge of participants subjected to an intervention consisting of five seminars on screening and preventive medicine, supplemented with a checklist as reminder. The control group, in contrast, did not receive the checklist, nor were encouraged to attend the seminars. Significant differences emerged post-intervention in terms of attitude and overall test results (both *p* < 0.05), with the intervention group demonstrating superior scores. It is noteworthy, that both groups, compared to their baseline, achieved a significant increase in overall score (*p* < 0.05). Cohen, Littenberg [22] further noted a strong correlation between improved test scores in the intervention group and their attendance at the seminars. Interestingly, seminar attendance did not translate into the use of preventive procedures, a finding that aligns with the research of Karuza, Calkins [26].

In contrast, Ngamruengphong, Horsley-Silva [66] focused solely on measurement of knowledge. The intervention group, which received additional education, a pocket card and monthly reminders showed a significant increase (*p* < 0.001) in knowledge about the HBV vaccination compared to baseline scores directly after the intervention. While there was a slight decline in scores for the intervention group after 6 months, their scores remained significantly higher than the control group, which received standard education (*p* < 0.001).

### Kirkpatrick level 3: behaviour

A total of seventeen studies investigated the behavioural changes among HCWs consequent to specific interventions [24, 25, 28, 30, 31, 33, 34, 37–40, 45, 47, 58, 59, 64, 66]. A common outcome utilized was the proportion of offered or ordered vaccines. We conducted an analysis on these outcomes, excluding the intervention category ‘other’ due to the heterogeneity of the interventions compared. The analysis revealed that general reminders and tailored reminders emerged as the most effective interventions when compared to usual care. Furthermore, education-only was least effective when compared to other interventions (Fig. 2).

#### General reminders versus usual care

We identified a single relevant study in this category Dubey, Mathew [47], which provided strong evidence for the superiority of general reminders over usual care. The authors found that while tetanus immunization decreased by 10.3% points in the control group, the group receiving checklist reminder registered an increase of 28.1% points. The pattern was replicated with rubella vaccinations, which exhibited a marginal decline of 0.4% points in the control group, but a substantial rise of 19.2% points in the intervention group.

#### Tailored reminder versus usual care

Six articles reported on this outcome level in this intervention contrast category [25, 31, 34, 37, 38, 45], of which five were published before 2000 [25, 31, 34, 37, 38]. Also, five of the six studies reported a significant effect of the tailored reminder system vs. usual care [25, 31, 37, 38, 45] and will be discussed below.

Flanagan, Doebbeling [25] investigated the impact of online reminders on vaccine ordering. Their findings suggested a significant increase only in ordered vaccines for physicians receiving a reminder for hepatitis vaccine (*p* = 0.004), while the difference was not significant for tetanus (*p* = 0.089), influenza (*p* = 0.320), pneumococcal (*p* = 1.000) and measles vaccines (*p* = 0.385).

McDonald, Hui [31] studied the role of computer reminders in enhancing the compliance with preventive care measures in primary care settings. The study reported on both the intention to administer influenza and pneumococcal vaccination, and actual response rate to an indication. While intention scores were high 4.2–4.4 in both study groups for influenza and pneumococcal vaccination, response rates to required influenza and pneumococcal vaccinations remained low in the control group (20% and 14%), whereas in contrast it increased up to 46% and 51% in the intervention group (both significant (*p* < 0.0001). There was a statistically significant relationship between intention and response rate in the intervention group (*p* = 0.03, r^2^ = 0.33), which was absent in the control group (*p* = 0.26, r^2^ = 0.12).

Schreiner, Petrusa [37] measured the effect of a paper reminder attached to the patient chart on pneumococcal vaccination compliance defined as offered or received vaccination. The increase was significant in the intervention group compared to the baseline (*p* < 0.001), but not compared to the control group. Additionally, a significant decrease in compliance was noted after a six months follow-up without reminders (*p* < 0.001).

Furthermore, Tang, LaRosa [38] measured influenza vaccination compliance with the influenza vaccine guidelines, defining it as any of the following: “*documentation that a clinician ordered the vaccine, counselled the patient about the vaccine, offered the vaccine to a patient who declined it, or verified that the patient had received the vaccine elsewhere*”. The study compared a computer-based patient record generating reminders with paper patient records devoid of reminders. The compliance of the computer-based system users increased significantly (*p* < 0.02), whereas paper record users’ compliance fell by 17% (*p* < 0.03) compared to the previous year. However, given that 27.9% of the paper records were missing, we decided to exclude this study from our analysis.

Finally, Dexter, Perkins [45] looked at the impact of reminders on preventive care in an inpatient setting, specifically on pneumococcal and influenza vaccination orders. The study found marked difference in vaccination orders, of 0.8-1% in the control compared to 35.8–51.4% in the study group using reminders (*p* < 0.001), with continued reminders maintaining increasing vaccination orders by 50–57% over 15 months following the study. The authors compared these findings with the results of an earlier study by Overhage, Tierney and McDonald [34], which found no significant impact of reminders. One difference between the two studies was that in Dexter’s study reminders in the form of prewritten orders were automatically and repetitively displayed on the screen, whereas in the Overhage’s study HCWs had to take action to see the recommendations. Additionally, Dexter’s study did not allow users to escape the reminder screen, and the default response was set to order the vaccination. Thus, the method of displaying tailored reminders to HCWs seems to play a role in its efficacy.

#### Tailored reminder versus non-education intervention

One article was identified in this category. In the study of Tape and Campbell [39] both the intervention and the control group received education. However, the intervention group also received a computerized tailored reminder, while the control group received a general reminder in the form of a paper flowsheet attached to the patient charts. The intervention group showed a significantly higher compliance rate for influenza (29.3% vs. 21.5%, *p* = 0.05), pneumococcal (11.3% vs. 4.7% *p* = 0.003), and tetanus vaccinations (5.6% vs. 2.6% *p* = 0.001) compared to the control group. There were no significant differences in compliance in relation to the format of the reminder (printed vs. digital).

#### Education only versus other intervention

Three articles were identified in this intervention category, comparing education with standing orders for nurses [24], physician reminders [24, 58] and one comparing education only intervention to a multicomponent intervention [28]. There was no evidence that education only would be more effective than other interventions.

Crouse, Nichol [24] compared standing orders, physician reminders and physician education in inpatient setting. Authors reported that the group with standing orders had the highest proportion of offered and registered vaccination (95% and 40%), followed by physician reminders (22% and 17%), and physician education (12% and 10%), *p* < 0.001 (Figs. 2 and 3). While physicians who received reminders or education offered fewer vaccination than nurses with standing orders, participants were more likely to accept vaccination from a physician than from a nurse.

Warner and Seleznick [58] compared education with education plus a reminder stamped and affixed to the medical charts. After six and twelve months the intervention group showed significantly higher pneumococcal vaccination documentation compared to the control group (85% vs. 65% after six months, *p* < 0.005, and 76% vs. 58% after twelve months, *p* < 0.05).

Kim, Kristopaitis [28] compared mailed educational materials to a multicomponent intervention that included the same educational materials, peer-comparison feedback, and academic detailing. The outcomes were mainly based on patient recall of being offered a vaccine. The recall for influenza, pneumococcal and tetanus vaccines being offered increased statistically significantly by 10, 31 and 8% points (*p* < 0.01) in the education only group. For influenza vaccine this increase did not differ significantly from the multicomponent intervention group (*p* = 0.86), whereas for pneumococcal and tetanus vaccinations, the increase was statistically significantly less than in the multicomponent intervention group (*p* = 0.02 and *p* < 0.01). Interestingly, according to medical record reviews there was no significant increase in the number of patients who were vaccinated, regardless of the intervention type (Fig. 3). The authors noted the limitation that the medical record may not accurately reflect the actions offered, but only the actions performed.

#### Multicomponent intervention versus usual care

Only one of two articles identified in this category reported a significant effect. Korn, Schlossberg and Rich [30] studied the effects on compliance with preventive care guidelines of a didactic seminar on health maintenance screening, biweekly chart review conferences with performance feedback, and a health maintenance checklist. They found a significant improvement in compliance with pneumococcal vaccination guidelines after intervention (*p* < 0.01), but not with the influenza vaccination guidelines. Nevertheless, the intervention group had a higher compliance with the influenza vaccination guidelines than the control group (*p* = 0.03). The study was not included in Fig. 2 because the effect measures could not be extracted nor calculated.

#### Other

Ornstein, Garr [33] studied the efficacy of reminders in addition to educational and administrative interventions, including quarterly audits and health maintenance flowchart. They found statistically significantly (*p* < 0.0001) higher increase in adherence to tetanus vaccination when education was supplemented with either patient (9.5% increase) or physician reminders (10.5%), or both (12%), compared to education only (3.8%).

Tierney, Hui and McDonald [40] reported on compliance with pneumococcal vaccinations using feedback reports and reminders. They found greater compliance among physicians receiving either reminders or feedback compared to controls (*p* < 0.01), but the effects were not additive.

Loskutova, Smail [64] compared a multicomponent intervention involving a clinical decision support, provider reminders, provider education, audit, feedback and improved documentation process to clinical decision support and provider reminders only. They found significant reductions in missed opportunities for influenza (-9.1% and -10.1%, both *p* < 0.0001) and pneumococcal vaccinations (-6.6% and -4.3%, both *p* < 0.0001) in both the intervention and the control groups. For herpes zoster vaccination, reductions in missed opportunities were significant only in the intervention group (-5.6%, *p* < 0.0001). Missed opportunities correlated with lower vaccination rates.

Winston, Lindley and Wortley [59] found low vaccination rates with a range of 1-18% and significant variation of patient refusal across hospitals despite the implementation of inpatient vaccination policies, including standing orders and physician reminders.

### Kirkpatrick level 4: results

#### Vaccination rate

Out of the 48 included studies included in the analysis 34 reported on changes in vaccination rates following the interventions [20–24, 26–29, 32, 35, 36, 41–44, 46, 48–57, 60–65, 67, 68, 70]. Outcomes on Kirkpatrick level 4 ‘results’ were reported by all intervention categories. The main study outcome was ‘vaccination rate’ (Fig. 3). The findings of the analysis indicate that multicomponent interventions and tailored reminders were the most effective interventions when compared to usual care (Fig. 3).

#### General reminders versus usual care

Two studies documented vaccination rates as a result of such [23, 44]. Neither of these studies found a significant difference between general reminders and usual care.

#### Tailored reminders versus usual care

Eight articles explored the outcome of a ‘tailored reminder system vs usual care’, all of which reported a significant result [29, 32, 35, 36, 60, 61, 63, 70]. Considering the varying study period, we conducted a subgroup-analysis based on publication year (before and in 2000 vs. after 2000). Six of these articles were published before 2000 [29, 32, 35, 36, 70].

McDonald, Hui and Tierney [32] reported that the intervention group’s influenza and pneumococcal vaccination rates were almost double that of the control group (*p* < 0.001). There were also fewer winter hospitalization (*p* < 0.01) and emergency room visits in the intervention group (*p* < 0.05).

A positive effect was similarly reported by Hutchison [70], noting significant increases in the intervention group following the introduction of computer reminders (*p* < 0.0001), while no change was seen in the control group.

In parallel, Rosser, Hutchison [35] and Rosser, McDowell and Newell [36] revealed that the randomized control group accomplished only 3.2% of the requisite tetanus vaccinations and 9.8% of the influenza vaccinations. In contrast this was higher in the group with physician reminders, with 22.8% and 22.9% respectively. Additionally, they studied the efficacy of written reminders sent to patients, and telephone reminders directed to patients. They found that these types of reminders boosted the proportion of vaccinations administered compared to the control group, as well. The highest rates were achieved when telephone reminders were used, 37% for influenza vaccination and 24% for tetanus vaccination. It should be noted that out of the 1471 patients allocated to the physician reminder group only 766 (52.1%) visited the practice during the year-long trial, consequently a large proportion of patients were not informed about vaccination.

Klein and Adachi [29] found poor pneumococcal vaccination rates, remaining at or below 20% after intervention. Despite this, significant differences were found between the intervention group and control group over two years (*p* < 0.001).

The three post-2000 studies consistently showed higher efficacy of computer reminders versus usual care, leading to increased vaccination uptake [60, 61, 63]. In these studies, the difference in vaccination rate between the tailored reminder and usual care groups post-intervention ranged from 3 to 12.7%.

Changolkar, Rewley [60] examined differences between physicians with handling varying patient volumes. The authors found a significant effect only in the group of physicians managing a higher workload (*p* = 0.01).

In the study by Loo, Davis [63] the use of automatic reminders showed a significantly better performance in influenza (*p* < 0.0.001) and pneumococcal (*p* = 0.04) vaccination rates than usual care.

Desai, Lu [61] investigated patient-level factors influencing pneumococcal vaccination among immunosuppressed patients. They reported that *“[physician] having received the point of care reminder”* significantly increased the probability of administering the vaccine (HR 3.58, 95% CI 2.46–5.20).

#### Tailored reminders vs. non-education intervention

In the intervention category ‘tailored reminders vs. other interventions than education’ two studies reported on vaccination rate as outcome [46, 63]. Efficacy of tailored reminders ranged from -23 to 31% and were mostly outranked by other non-education interventions like standing order.

Dexter, Perkins [46] found that standing orders resulted in statistically higher rate of vaccine administration than physician reminders for influenza and pneumococcal vaccinations (*p* < 0.001). This was mainly due to the relatively low physician compliance with the automatic pop-up messages with orders on the required vaccines.

Loo, Davis [63] showed similar statistically significant increase in influenza and pneumococcal vaccinations in both reminder only and reminders plus panel manager groups compared to the control group. The panel manager assisted both patients and physicians in completing the four targeted preventive healthcare procedures including influenza vaccinations and pneumococcal vaccination.

#### Comparison of different tailored reminders

Six articles compared the efficacy of different types of interventions [21, 35, 36, 41, 42, 51] of these, the article by Rosser, McDowell and Newell [36] did not measure effectiveness per reminder type for a given preventive care procedure.

Chambers, Balaban [21] delved into the impact of the frequency of reminders displayed. The study’s physicians were split into three groups: (1) those who received reminders for every eligible patient, (2) those who received reminders for half of the eligible patients, and (3) those who did not receive reminders at all. Patients whose physicians received reminders for every eligible patients were more likely to get the influenza vaccination compared to patients of physicians who did not receive reminders (*p* < 0.001). Interestingly, physicians who received intermittent reminders were less likely to have vaccinated eligible patients for whom they did not receive a reminder, compared to those physicians who did not receive any reminders (*p* < 0.001). This suggests a dependency of physicians on the reminders.

Moreover, Turner, Waivers and O’Brien [42] discovered that a dual physician reminder, computer-generated reminder attached to the patient chart coupled with patient-carried reminder cards resulted in an increased influenza vaccination rate compared to computer-generated reminders alone (47% vs. 29%, *P* < 0.002), but no difference was observed in pneumococcal vaccination rates. However, when authors repeated the experiment a few years later by comparing groups receiving only the computer-generated reminder or patient-carried cards, no difference was found, which was explained by not adding the patient-cards as a completion to computer-generated reminders.

Rosser, Hutchison [35] and Rosser, McDowell and Newell [36] studied the effect of different reminder types on the use of preventive services, among which influenza and tetanus vaccinations; (1) physician reminder, (2) letter reminder to patient, and (3) telephone reminder to patient. In the physician reminder-group 22.9% and 22.8% of the required influenza and tetanus vaccines were performed, this was 35.2% and 30.6% in the letter reminder-group. In the telephone reminder group 37.0% of the required influenza vaccines and 24.0% of the tetanus vaccines were given. Moreover, Rosser, Hutchison [35] found that letter reminders were more effective than telephone reminders (*p* = 0.00013) and physician reminders (*p* < 0.00001) in increasing the tetanus vaccination rate.

#### Education only versus other intervention

Two studies investigated ‘education only’ interventions versus other interventions while reporting on vaccination uptake [24, 28]. One compared ‘education only’ interventions with physician reminders and standing order procedures [24] (see level 3), while another compared education to a multicomponent intervention [28]. Kim, Kristopaitis [28] found significant changes when calculations were based on patient recall. However medical record reviews for influenza vaccination did not reveal any increase in the proportion of patients who were offered the procedure.

#### Multicomponent intervention versus usual care

Thirteen studies examined multicomponent interventions versus usual care, reporting on vaccination rate as an outcome [20, 22, 26, 27, 43, 48, 50, 53, 55, 65, 67–69]. Almost all articles– with the exception of Kerse, Flicker [27]– indicated a distinct effect of complex educational interventions versus usual care, the difference ranged from -3.5 to 37.2%. The applied approaches to initiate changes varied across studies; some described complex interventions with pre-specified building blocks [43, 65, 69], while others emphasised group consensus and encouraged practices to find their own solutions for improving vaccination rates [20, 26, 50, 55, 67].

In the study by Cohen, Littenberg [22] combining seminars with checklists affixed to patient charts resulted in statistically significant difference in the delivery of influenza immunization (*p* < 0.001).

Nowalk, Nutini [67] found that group consensus and commitment may be crucial elements of successful provider-based interventions. They observed higher vaccination coverage in those two intervention sites where medical staff were committed to the adaptation towards standing order procedures. However, at the third site, the medical assistants felt to be imposed to increase their workload and were reluctant to taking on additional responsibilities. This resulted in no change in vaccination rates [67].

Karuza, Calkins [26] also emphasized the social context and group dynamics of introducing organizational changes in medical practice. Researchers implemented a small-group consensus process, which included an educational session followed by group discussions and a group commitment to implement organizational changes. The most common solution was mailing patient reminders, followed by setting up a poster in the waiting room, and implementing a system of chart reminders. The influenza vaccination rate was higher in the intervention group compared to the control group receiving placebo intervention (62.4% vs. 46.5%, *p* < 0.001).

A two-year follow-up study which applied small-group consensus process was conducted by Calkins, Katz [20]. After the small-group consensus process, no further centralized effort was taken to boost physicians’ vaccination performance. Each practice further discussed and decided amongst themselves three procedures they wanted to implement to increase influenza vaccination rates. These procedures consisted of chart reminders, patient education materials, mailed reminder, staff education, organizational changes. The vaccination rate increased with 11.5% (*p* < 0.01) in the intervention practices after the small-group consensus process was conducted. After two-year follow-up, additional increases of 5.3–6.5% points were found in the intervention practices.

Shultz, Malouin [68] implemented an automatic reminder system, which followed the achievement of consensus to maximize the support of the medical staff. However, after the active surveillance period, the vaccination performance decreased in the active study group and an increase was seen in the control group (decrease of -7% vs. increase of 8%, respectively).

McGreevy, McGowan [65] examined a multicomponent intervention heavily relying on non-physician care-team members. Despite seeing an increase in vaccination rates and carry-over effect outside the study population they also observed a decrease post-study, mostly due to nurses discontinuing chart reviews before patient visits. They also noted that communication issues were often the root cause of missed opportunities for vaccination.

Solberg, Kottke [55], who conducted a complex quality improvement project found a significant difference in the increase in pneumococcal vaccination: 17.2% points in the intervention group and 0.3% in the control group (*p* = 0.003). A carryover effect was also in tetanus immunization, which was not targeted during the project.

#### Other

Nine studies were included in this review that did not fit any of the earlier mentioned intervention categories, but reported on vaccination rates [24, 49, 52, 54, 56, 57, 59, 62, 64] (see level 3 for the study by Crouse, Nichol [24]).

Kiefe, Allison [49] compared a multicomponent intervention plus performance feedback with the same multicomponent intervention complemented with achievable benchmark feedback. The group receiving achievable benchmark feedback showed an 18% point increase in vaccination compared to six in the other group (*p* < 0.001).

Quinley and Shih [52] showed that physicians receiving mailed feedback on the rate of the pneumococcal vaccination coverage rate of the previous year plus educational materials, offers of assistance, and telephone follow-up had significantly higher rates of pneumococcal vaccination coverage as compared to the group receiving only mailed feedback.

Siriwardena [54] compared a multicomponent educational intervention including audit feedback and written guidance compared to receiving only audit feedback and written guidance. Pneumococcal vaccination rates improved significantly in the intervention group for two out of three specific patient populations, but not for older adults in general. No significant differences were found between the intervention and control group for influenza vaccination. The lack of more between-group differences could have been influenced by a concurrent nationwide health education campaign.

Stevenson, McMahon [56] evaluated the long-term care facilities’ intervention strategies for improving pneumococcal vaccination rates among residents. Overall, the vaccination rates increased up to 69–84% from the baseline range of 26–53%, all changes being statistically significant (*p* < 0.001). They found that standing orders and physician reminders appeared to be the most efficient solutions. The authors concluded that collaboration with quality-improvement organizations helped facilities structure and organize their programs.

Trick, Das [57] compared different combinations of standing orders. The group with standing orders plus opt-out orders for physicians achieved the highest influenza vaccination rate.

Winston, Lindley and Wortley [59] found that despite implementing inpatient vaccination policies, which included standing orders and physician reminders, vaccination rates remained low in community hospitals (range: 1-18%).

In the study of Hohmann, Hastings [62], changes in pneumococcal vaccinations were statistically significant in the intervention group compared to baseline (*p* = 0.007), but not when compared to the control group due to lack of power. No significant changes were found in the intervention group for herpes zoster vaccination rates.

Loskutova, Smail [64] found no significant differences between clinical decision support and provider reminders only and a multicomponent intervention including these clinical decision support, provider reminders, except for pneumococcal vaccination in high-risk adults below 65 years. Here, clinical decision support provider reminders only performed significantly better (*p* = 0.001).

#### Organizational change

Two studies reported another outcome ‘organizational changes’ among level 4 ‘results’ [43, 62].

van Essen, Kuyvenhoven and de Melker [43] investigated the effect of guideline adoption in intervention regions compared to control regions. They studied several aspects of practice, such as registering high risk patients, sending e-mail prompts, stocking influenza vaccine, organizing special vaccination hours, and delegating vaccinations to practice assistant. Significant improvements were seen in the intervention regions for having influenza vaccine in stock (*p* < 0.001), using mail prompts (*p* < 0.001), holding special vaccination hours (*p* < 0.05).

Hohmann, Hastings [62] studied structural and process activities, like developing an evaluation plan and setting objectives for each vaccine provided, following a 6-month immunization program targeting pharmacies. Statistically significant results were observed in the intervention group between baseline and post-intervention for pneumococcal vaccine (*p* = 0.007) and total vaccine doses (*p* = 0.014). No statistically significant differences were observed between the control group, which received only information on immunization update, and the intervention group.

### Risk of bias

**Fig. 4:**
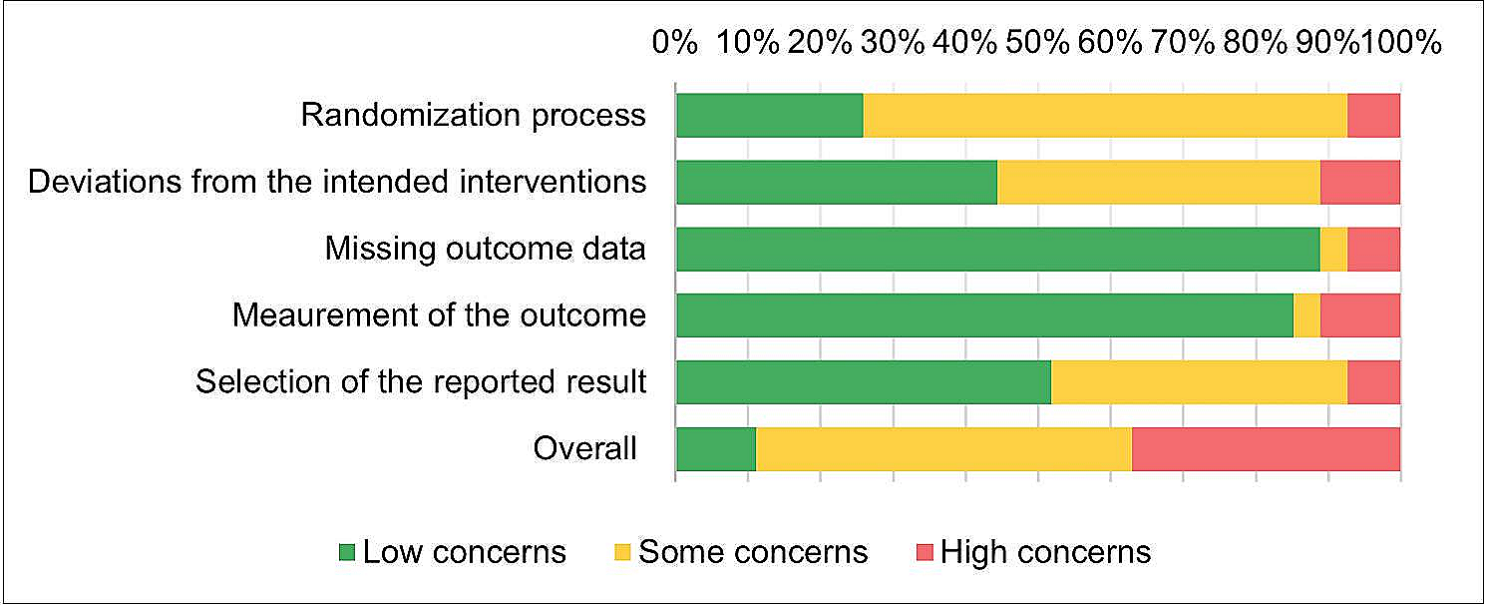
Presence of risk of bias items across randomized controlled trials

Risk of bias was evaluated for 27 RCTs and 21 non-randomized studies included in this review, to assess methodological limitations of included studies. Three RCTs were assessed as low risk of bias [23, 47, 55], 14 gave reason for some concern [20, 21, 26, 28, 34–36, 40, 44–46, 49, 52, 54, 62, 66], and 10 were associated with high risk of bias [22, 25, 27, 29, 31–33, 41, 42, 50, 51]. The relatively high share of studies with some concerns or high risk of bias could be attributed to a lack of information in some of the domains relevant for the risk of bias assessment (Table 2; Fig. 4).

**Table 2:**
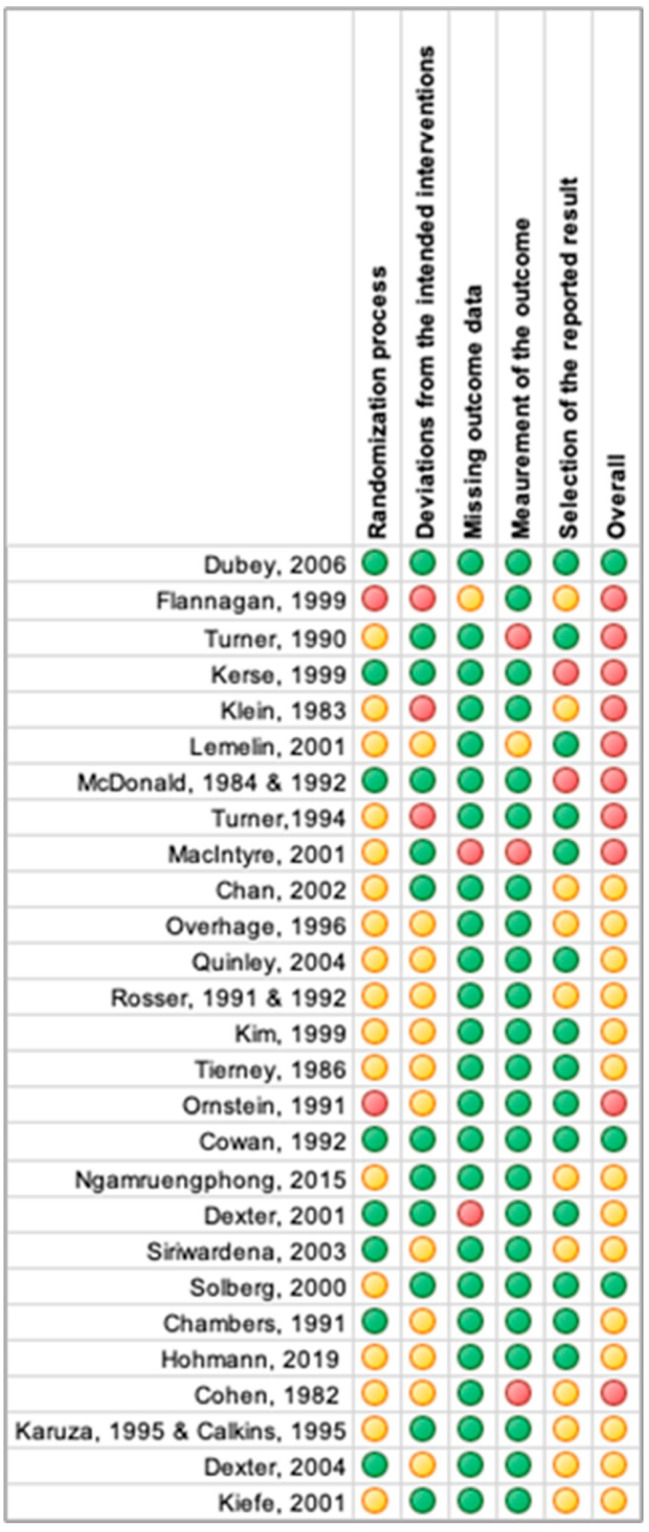
Risk of bias summary of randomized controlled trials

**Fig. 5:**
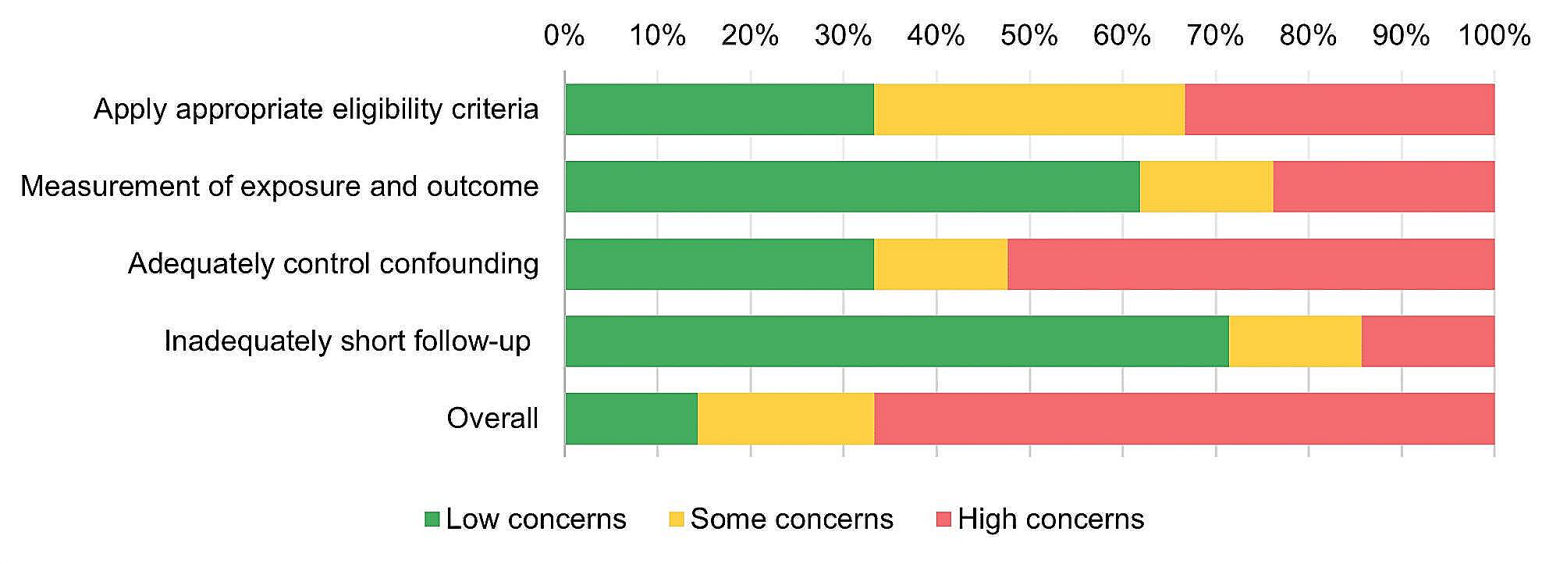
Presence of risk of bias items across non-randomized studies

The 21 non-randomized studies were assessed for bias by using the Grading of Recommendations Assessment, Development and Evaluation (GRADE) guideline [19, 71]. According to this assessment, three studies were associated with low risk of bias [60, 63, 68], four gave reason for some concern [30, 43, 48, 61], and fourteen studies were judged to be at high risk of bias [24, 37–39, 53, 56–59, 64, 65, 67, 69, 70]. The high risk of bias in the majority of studies was due to the predefined decision of the reviewers that if a study lacks description of methods to control for confounding, the overall risk of bias for the studied outcome would be judged high (Table 3; Fig. 5).

**Table 3:**
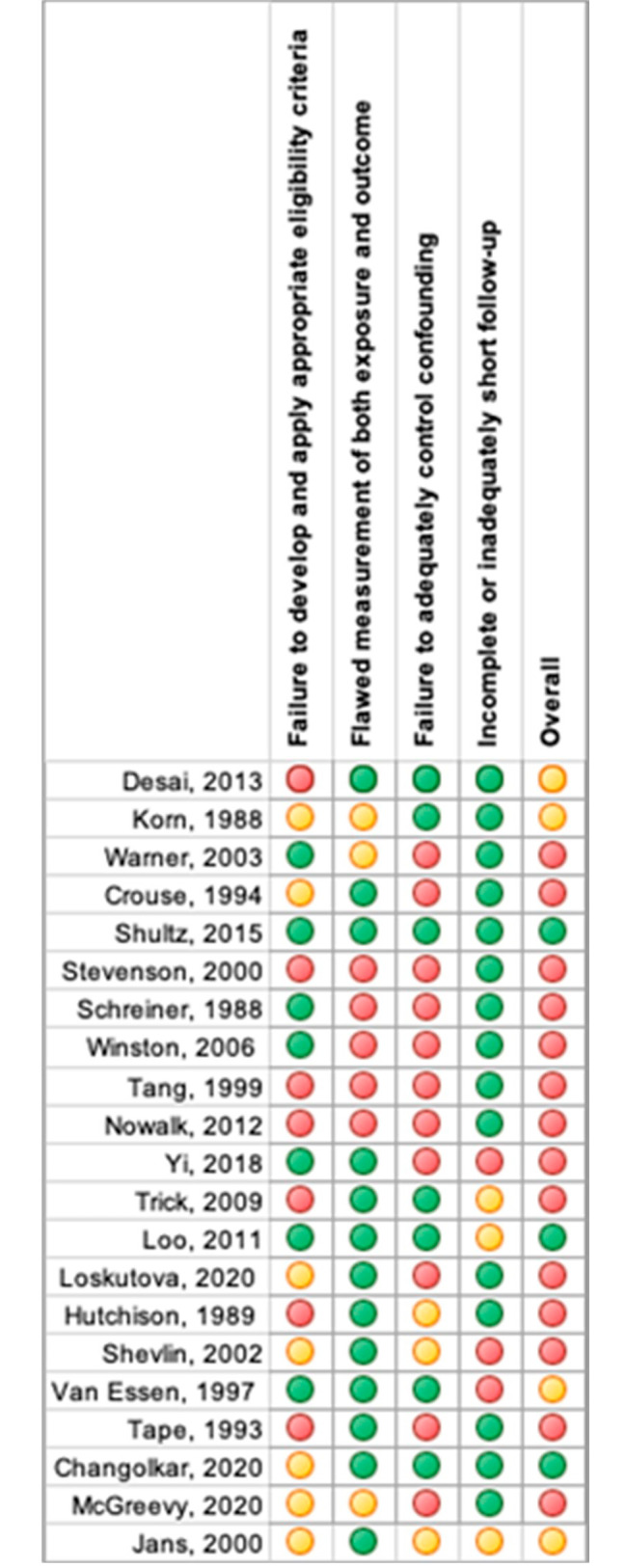
Risk of bias summary of non-randomized studies

## Discussion

We found that two out of six articles reporting on the learning-level (Kirkpatrick level 2), reported positive changes in HCWs’ attitude or knowledge due to the multicomponent intervention. When considering behaviour changes of HCWs (Kirkpatrick level 3), the study found that tailored reminders were most effective, followed by general reminders. Education alone, such as lectures, was not effective in changing HCWs’ behaviour when compared to other interventions. Regarding interventions that effectively increased vaccination rates (Kirkpatrick level 4), multicomponent interventions were most effective compared to usual care, followed by tailored reminders. However, tailored reminders were often less effective than other interventions such as standing orders or patient reminders. Again, standalone education was often ineffective when compared to other interventions.

Our finding that education only interventions were less effective than other interventions was contrary to our expectation. We expected education to have a positive effect on HCW’s vaccine knowledge. Previous studies found that the extent of vaccine knowledge among HCW vaccination recommending was positively associated with HCW willingness to recommend vaccines [10] and intention to vaccinate [72]. However, we identified one study where HCW knowledge increased due to education, yet there was no effect on the vaccination rates [22]. Moreover, in another study there was no increase in knowledge, yet the vaccination rates increased [26]. This might indicate that perhaps other factors play a role in the relationship between education, knowledge and vaccination rates. More research should be conducted to understand this relationship.

Our results regarding reminder systems are in line with previously conducted reviews [13–15]. Despite these previously conducted reviews not considering reminder systems as an educational intervention, this study viewed reminders as a form of learning through repetition [17]. The theory is that repetitive exposure to reminders would engrain the information deeper into the minds of HCWs, reminding them to offer vaccination. However, two studies indicated that HCPs may become dependent on these reminders [21, 37]. Regarding the effectiveness of education-only interventions, our findings differ slightly from the results found by Lau, Hu [13] who found an association between HCW education and improved pneumococcal vaccination rates. However, education was found to be ineffective for influenza vaccination rates.

Our review contributes to existing literature by adopting a broader scope in evaluating interventions including reminders, quality improvement projects, audit & feedback activities and encompassing various types of adult vaccinations. Also, our categorization of interventions with two or more components as ‘multicomponent’ is a strength of our study. Only the review conducted by Ndiaye, Hopkins [14] specifically investigated the effectiveness of studies composed of multiple intervention types. However, whereas the review of Ndiaye, Hopkins [14] identified several multicomponent intervention studies, only one focused solely on different provider-based interventions. Moreover, our systematic review has a strength including studies of an unrestricted time span covering up to 40 years. As both vaccines and vaccination policies have undergone substantial changes over time, we performed sub-group analyses by time period, when the data permitted. However, this was only feasible for studies describing tailored reminder systems, and no significant difference was found between studies published before versus after 2000.

There were some limitations in the studies included in this review. We hoped to find in-depth descriptions of the educational interventions, including content of lectures and use of different educational working methods, but the descriptions in the identified studies were generally rough. Nevertheless, we could differentiate between the fundamental building blocks of the described interventions, categorize them (e.g., reminder systems; education only; multicomponent interventions, etc.) and analyse their efficacy on the four measured levels of the Kirkpatrick model.

Studies focusing specifically on older adults over 50 years were scarce. Therefore, studies focusing on older adults and risk-groups in more general terms were also included. Moreover, many of the included studies aimed to increase vaccine coverage among older adults, rather than focusing on the HCW-patient dialogue necessary for older adults to make an informed decision on vaccination behaviour. Moreover, we noticed that only a few studies considered how HCWs perceived the intervention, which could influence the efficacy of these interventions. High risk of bias was found in the majority of studies based on RoB2 and GRADE risk of bias assessments. The included studies were distributed unevenly by vaccination type and geography. Few papers included herpes zoster vaccine and most of them were from Western countries, which might limit the generalizability of the results.

## Conclusions

Our review highlights the importance of tailored reminders and multicomponent interventions for practical implications. Implementing tailored reminders and multicomponent interventions in primary care and hospitals could improve the ability of HCWs to engage into a dialogue on vaccines with their older adult patients. Solely relying on education through lectures is not effective; however, when combined with other interventions, as in multicomponent interventions, education through lectures can yield positive outcomes.

### Electronic supplementary material

Below is the link to the electronic supplementary material.

Supplementary Material 1

Supplementary Material 2

## Data Availability

The search strategies can be found in the appendices, as well as the list of collected data items and the GRADE checklist for nonrandomized studies, including the agreements made between the authors regarding assessment of studies. The research protocol was registered with PROSPERO and can be found under registration number: CRD42020180165. The datasets used and/or analyzed during the current study are available from the corresponding author on request.

## Abbreviations

HCW: Healthcare workers
RoB 2: Revised Cochrane risk-of-bias
RCTs: Randomized Controlled Trials
GRADE: Grading of Recommendations Assessment, Development and Evaluation
PROSPERO: International Prospective Register of Systematic Reviews
