# Correction: Effectiveness of educational interventions for healthcare workers on vaccination dialogue with older adults: a systematic review

**DOI:** 10.1186/s13690-024-01278-5

**Published:** 2024-04-11

**Authors:** Manuela Dominique Wennekes, Tímea Almási, Renske Eilers, Fruzsina Mezei, Zsuzsanna Ida Petykó, Aura Timen, Zoltán Vokó, VITAL Consortium

**Affiliations:** 1 Centre for Infectious Disease Control, National Institute of Public Health and the Environment (RIVM), Bilthoven, The Netherlands; 2 Athena Institute, VU University Amsterdam, Amsterdam, The Netherlands; 3 Syreon Research Institute, Budapest, Hungary; 4 Department of Primary and Community Care, Radboud University Medical Center, Nijmegen, The Netherlands; 5 Center for Health Technology Assessment, Semmelweis University, Budapest, Hungary

**Correction: Arch Public Health 82, 34 (2024).**

10.1186/s13690-024-01260-1.

Following publication of the original article [1], the authors reported an error in Abstract section. The last three words “rates, attitude, knowledge” in Abstract conclusion part were wrongly placed and should be placed as the last three words in Keywords section.

The original article [1] has been updated.
