# Supplementary material for: Effectiveness of educational interventions for healthcare workers on vaccination dialogue with older adults: a systematic review
Source: Arch Public Health. 2024 Mar 12;82:34. doi: 10.1186/s13690-024-01260-1 (PMC10929108; doi:10.1186/s13690-024-01260-1)
Supplement: Supplementary file 1 — Supplementary Material 1 [file 13690_2024_1260_MOESM1_ESM.docx]

**Appendices**

Appendix 1: Search strategy MEDLINE (via PubMed)

Result: 1889 hits (1^st^ of April 2020)

1. health personnel[mh] OR geriatric nursing[mh]

2. healthcare[tiab] OR health-care[tiab] OR health care[tiab] OR social[tiab] OR hospital[tiab] OR clinic[tiab]

3. provider[tiab] OR providers[tiab] OR staff[tiab] OR personnel[tiab] OR specialist[tiab] OR specialists[tiab] OR professional[tiab] OR professionals[tiab] OR worker[tiab] OR workers[tiab] OR profession[tiab]

4. 2 AND 3

5. general practitioner[tiab] OR general practitioners[tiab] GP[tiab] OR nurse[tiab] OR nurses[tiab] OR nursing[tiab] OR physician[tiab] OR physicians[tiab] OR doctor[tiab] OR doctors[tiab] OR family practitioner[tiab] OR family practitioners[tiab] OR pharmacist[tiab] OR pharmacists[tiab] OR clinician[tiab] OR clinicians[tiab] OR paramedic*[tiab]

6. 4 OR 5

7. 1 OR 6

8. education[mh] OR teach-back communication[mh] OR health communication[mh] OR reminder system[mh]

9. educate[tiab] OR educates[tiab] OR educated[tiab] OR educating[tiab] OR education[tiab] OR educational[tiab] OR train[tiab] OR trains[tiab] OR trained[tiab] OR training[tiab] OR teach[tiab] OR teaches[tiab] OR taught[tiab] OR teaching[tiab] OR learn[tiab] OR learns[tiab] OR learned[tiab] OR learning[tiab] OR instruct[tiab] OR instructs[tiab] Or instructed[tiab] OR instructing[tiab] OR instruction[tiab] OR feedback[tiab] OR remind*[tiab] OR academic detailing[tiab] OR “peer comparison” [tiab] OR competition[tiab] OR outreach[tiab] OR workshop[tiab] OR seminar[tiab] OR quality improvement[tiab] OR quality assurance[tiab]

10. 8 OR 9

11. vaccines[mh] OR immunization[mh]

12. immunity[tiab] OR immunization[tiab] OR immunizations[tiab] OR immunizational[tiab] OR immunisation[tiab] OR immunisations[tiab] OR vaccin*[tiab] OR inoculat*[tiab]

13. 11 OR 12

14. adult[mh] NOT young Adult[mh]

15. health services for the aged[mh] 16. 14 OR 15

17. “older adult”[tiab] OR “older adults”[tiab] OR elderly[tiab] OR middle aged[tiab] OR “old age”[tiab] OR

senior[tiab] 18. 16 OR 17

19. matern*[ti] OR pregnan*[ti] OR parent*[ti] OR mother*[ti] OR father*[ti] OR pediatric*[ti] OR child[ti] OR childhood[ti] OR children[ti] OR toddler*[ti] OR infant*[ti] OR "young adult"[ti] OR "young adults"[ti] OR tuberculosis[ti] OR poliomyelitis[ti] OR mumps[ti] OR "cross-sectional"[ti]

20. qualitative research[majr]

21. cross-sectional studies[majr] 22. 19 OR 20 OR 21

23. 7 AND 10 AND 13 AND 18

24. 23 NOT 22

Searching query for copy and paste:

Search (((((((((((health personnel[mh] OR geriatric nursing[mh]))) OR (((((healthcare[tiab] OR health-care[tiab] OR health care[tiab] OR social[tiab] OR hospital[tiab] OR clinic[tiab])) AND (provider[tiab] OR providers[tiab] OR staff[tiab] OR personnel[tiab] OR specialist[tiab] OR specialists[tiab] OR professional[tiab] OR professionals[tiab] OR worker[tiab] OR workers[tiab] OR profession[tiab]))) OR (general practitioner[tiab] OR general practitioners[tiab] GP[tiab] OR nurse[tiab] OR nurses[tiab] OR nursing[tiab] OR physician[tiab] OR physicians[tiab] OR doctor[tiab] OR doctors[tiab] OR family practitioner[tiab] OR family practitioners[tiab] OR pharmacist[tiab] OR pharmacists[tiab] OR clinician[tiab] OR clinicians[tiab] OR paramedic*[tiab])))) AND (((education[mh] OR teach-back communication[mh] OR health communication[mh] OR reminder system[mh])) OR (educate[tiab] OR educates[tiab] OR educated[tiab] OR educating[tiab] OR education[tiab] OR educational[tiab] OR train[tiab] OR trains[tiab] OR trained[tiab] OR training[tiab] OR teach[tiab] OR teaches[tiab] OR taught[tiab] OR teaching[tiab] OR learn[tiab] OR learns[tiab] OR learned[tiab] OR learning[tiab] OR instruct[tiab] OR instructs[tiab] Or instructed[tiab] OR instructing[tiab] OR instruction[tiab] OR feedback[tiab] OR remind*[tiab] OR academic detailing[tiab] OR “peer comparison”[tiab] OR competition[tiab] OR outreach[tiab] OR workshop[tiab] OR seminar[tiab] OR quality improvement[tiab] OR quality assurance[tiab]))) AND (((vaccines[mh] OR immunization[mh])) OR (immunity[tiab] OR immunization[tiab] OR immunizations[tiab] OR immunizational[tiab] OR immunisation[tiab] OR immunisations[tiab] OR vaccin*[tiab] OR inoculat*[tiab]))) AND (((((adult[mh] NOT young Adult[mh])) OR health services for the aged[mh])) OR (“older adult”[tiab] OR “older adults”[tiab] OR elderly[tiab] OR middle aged[tiab] OR “old age” [tiab] OR senior[tiab])))) NOT (((matern*[ti] OR pregnan*[ti] OR parent*[ti] OR mother*[ti] OR father*[ti] OR pediatric*[Title] OR child[ti] OR childhood[ti] OR children[ti] OR toddler*[ti] OR infant*[ti] OR "young adult"[ti] OR "young adults"[ti] OR tuberculosis[ti] OR poliomyelitis[ti] OR mumps[ti] OR cross-sectional[ti] OR qualitative research[majr] OR cross-sectional studies[majr])))

Appendix 2: Search strategy Scopus

Result: 1614 hits (1^st^ of April 2020)

1. TITLE-ABS-KEY ( "general practitioner" OR gp OR nurs* OR physician OR doctor OR "family practitioner" OR pharmacist OR clinician OR paramedic* )

2. TITLE-ABS-KEY ( healthcare OR health-care OR "health care" OR social OR hospital OR clinic)

3. TITLE-ABS-KEY ( provider OR staff OR personnel OR specialist OR professional OR worker OR profession)

4. 2 AND 3

5. 1 OR 4

6. TITLE-ABS-KEY ( educate OR educates OR educated OR educating OR education OR educational OR train OR trains OR trained OR training OR teach OR teaches OR taught OR teaching OR learn OR learns OR learned OR learning OR instruct OR instructs OR instructed OR instructing)

7. TITLE-ABS-KEY ( instruction OR feedback OR remind* OR "academic detailing" OR "peer comparison" OR competition OR outreach OR workshop OR seminar OR "quality improvement" OR "quality assurance" )

8. 6 OR 7

9. TITLE-ABS-KEY ( immunity OR immunization OR immunizational OR immunisation OR vaccin* OR inoculat*)

10. TITLE-ABS-KEY ( elderly OR middle AND aged OR "old age" OR senior OR old AND adult)

11. TITLE ( matern* OR pregnan* OR parent* OR mother* OR father* OR pediatric* OR child* OR toddler OR "young adults" OR tuberculosis OR poliomyelitis OR mumps OR "cross-sectional")

12. 5 AND 8 AND 9 AND 10

13. 12 AND NOT 11

Searching query for copy and paste:

( ( ( TITLE-ABS-KEY ( "general practitioner" OR gp OR nurs* OR physician OR doctor OR "family practitioner" OR pharmacist OR clinician OR paramedic* ) ) OR ( ( TITLE-ABS-KEY ( healthcare OR health-care OR "health care" OR social OR hospital OR clinic ) ) AND ( TITLE-ABS-KEY ( provider OR staff OR personnel OR specialist OR professional OR worker OR profession ) ) ) ) AND ( ( TITLE-ABS-KEY ( educate OR educates OR educated OR educating OR education OR educational OR train OR trains OR trained OR training OR teach OR teaches OR taught OR teaching OR learn OR learns OR learned OR learning OR instruct OR instructs OR instructed OR instructing ) ) OR ( TITLE- ABS-KEY ( instruction OR feedback OR remind* OR "academic detailing" OR "peer comparison" OR competition OR outreach OR workshop OR seminar OR "quality improvement" OR "quality assurance" ) ) ) AND ( TITLE-ABS-KEY ( immunity OR immunization OR immunizational OR immunisation OR vaccin* OR inoculat* ) ) AND ( TITLE-ABS-KEY ( elderly OR middle AND aged OR "old age" OR senior OR old AND adult ) ) ) AND NOT ( TITLE ( matern* OR pregnan* OR parent* OR mother* OR father* OR pediatric* OR child* OR toddler OR "young adults" OR tuberculosis OR poliomyelitis OR mumps OR "cross-sectional" ) )

Appendix 3: Search strategy Cochrane library

Result: 143 hits (19^th^ of May 2020)

1. ( "general practitioner" OR gp OR nurs OR physician OR doctor OR "family practitioner" OR pharmacist OR clinician OR paramedic) OR ((healthcare OR health-care OR "health care" OR social OR hospital OR clinic) AND (provider OR staff OR personnel OR specialist OR professional OR worker OR profession))

2. educat OR train OR teach OR learn OR instruct OR feedback OR remind OR "academic detailling" OR "peer comparison" OR competition OR outreach OR workshop OR seminar OR "quality improvement" OR "quality assurance"

3. immunit OR vaccin OR inoculat

4. elderly OR senior OR old age OR older adult OR middle aged OR old AND adult

5. matern OR pregnan OR parent OR mother OR father OR pediatric OR child OR childhood OR toddler OR "young adults" OR tuberculosis OR poliomyelitis OR mumps OR cross-sectional

Searching query for copy and paste:

("general practitioner" OR gp OR nurs OR physician OR doctor OR "family practitioner" OR pharmacist OR clinician OR paramedic) OR ((healthcare OR health-care OR "health care" OR social OR hospital OR clinic) AND (provider OR staff OR personnel OR specialist OR professional OR worker OR profession)) in Title Abstract Keyword AND educat OR train OR teach OR learn OR instruct OR feedback OR remind OR "academic detailling" OR "peer comparison" OR competition OR outreach OR workshop OR seminar OR "quality improvement" OR "quality assurance" in Title Abstract Keyword AND immunit OR vaccin OR inoculat in Title Abstract Keyword AND elderly OR senior OR old age OR older adult OR middle aged OR old AND adult in Title Abstract Keyword NOT matern OR pregnan OR parent OR mother OR father OR pediatric OR child OR childhood OR toddler OR "young adults" OR tuberculosis OR poliomyelitis OR mumps OR cross-sectional in Record Title

- (Word variations have been searched)

Appendix 4: Search strategy grey literature

We searched the following grey literature sources:

- European Disease Control (ECDC)
- World health Organization (WHO)
- OpenGrey:

Searches OpenGrey http://www.opengrey.eu/

AND is the default boolean between terms.

- Vaccin* adult* 155 results

- Immuniz* adult* 12 results

- Immunis* adult* 21 results

- Vaccin* elder* 14 results

- Immuniz* elder* 1 result

- Immunis* elder* 4 results

Total = 207 results

- Grey Literature Report

Searches Grey Literature Report http://www.greylit.org/

AND is the default boolean between terms. Truncation automatically from six characters and up.

- Vaccin adults 26 results

- immunization adults 24 results

- immunisation adults 1 result

- Vaccin elderly 6 results

- Immuniz elderly 0 results

- Immunis elderly 0 results

Total = 57 results

Appendix 5: List of collected data items

| **Data domain** | **Data collected** |
| --- | --- |
| Study details | - First author, year - Title - Sponsorship Source - Conflict of interest - Country / geographical location - Healthcare setting - Comments - Research question / aim - Main conclusion - Author’s reasoning why the study was / was not successful |
| Author’s contact details | - Author’s name - Email - Address - Institution (from all authors) |
| Methods | - Study design - Mixed methods study? - Total study period - Type of addressed vaccine(s) - Pre-specified outcomes |
| Reference population of health care workers | - Inclusion and exclusion criteria - Group differences at baseline - Number of included HCWs / clinics - Mean age - Percentage of females |
| Reference population of target group (patients) | - Inclusion and exclusion criteria - Group differences at baseline - Number of included patients - Mean age - Percentage of females |
| Intervention | - Name of intervention(s) - Short description of intervention - Who provided it? - Where was it provided? - Drop-out and/or omission rate (missing data) - Description of intervention - Didactical methods used in the intervention - Educational content of the intervention - Reminding elements - Other |
| Outcomes (extracted separately for before and after data) | - Outcome name (i.e. influenza vaccination rates) - Outcome type(i.e. change in vaccination coverage) - Time-point - Patient population type (i.e. eligible patients) - Number of participants to whom data refers (denominator) - Scale/Range - Value (%, n, mean value, OR,RR, etc.) - Notes (i.e. statistical method and significance level, method of adjusting) |

Heterogeneity of denominators used for calculating vaccination rates

When estimating the proportion of vaccinated patients, studies may differ in their applied patient pool. Proportion measures are highly sensitive to the applied denominator, as at a constant number of performed vaccinations, the smaller the patient pool is, the higher the vaccination rate we get. Therefore, we registered the ‘patient population type’ into the data extraction table according to the following categories:

- All registered patients in the medical database
- Eligible patients (with indication, no contraindication and not vaccinated)
- Attending patients (not necessarily being eligible for vaccination)
- Eligible AND attending patients (eligible patients who visited the physicians)

As our narrative synthesis is based on comparing the absolute changes on the different intervention arms and does not aim for a cross-study comparison of absolute vaccination rates, we will not stratify our results according to the above mentioned categories. However, we registered the type of patient pool used in our data extraction table in case future researchers intend to perform further analyses.

Appendix 6: GRADE checklist nonrandomized studies

| 1. Failure to develop and apply appropriate eligibility criteria (inclusion of control population) | Under- or over-matching in case-control studies |
| --- | --- |
|  | Selection of exposed and unexposed in cohort studies from different populations |
| Reasoning/relevant part from the article | |
| 2. Flawed measurement of both exposure and outcome | Differences in measurement of exposure (e.g. recall bias in case-control studies) |
|  | Differential surveillance for outcome in exposed and unexposed in cohort studies |
| Reasoning/relevant part from the article | |
| 3. Failure to adequately control confounding | Failure of accurate measurement of all known prognostic factors |
|  | Failure to match for prognostic factors and/or adjustment in statistical analysis |
| Reasoning/relevant part from the article | |
| 4. Incomplete or inadequately short follow-up | Especially within prospective cohort studies, both groups should be followed for the same amount of time. |
| Reasoning/relevant part from the article | |
| 5. Overall judgment | |
| Reasoning/relevant part from the article | |

Agreements for assessment of the risk of bias in non-randomized studies:

- In case there is a ”failure to adequately control confounding” we will automatically assign high risk of bias for the given outcome. When making the overall judgement, provide a detailed description why you decide on low/high/some concerns, this enhances transparency.
- Incomplete or inadequately short follow-up: If the authors themselves state this as a limitation, and for example when the follow-up end before the flu season has ended (in case of the intervention targeting the uptake of the flu vaccine)
