# Supplementary material for: Effectiveness of educational interventions for healthcare workers on vaccination dialogue with older adults: a systematic review
Source: Arch Public Health. 2024 Mar 12;82:34. doi: 10.1186/s13690-024-01260-1 (PMC10929108; doi:10.1186/s13690-024-01260-1)
Supplement: Supplementary file 2 — Supplementary Material 2 [file 13690_2024_1260_MOESM2_ESM.docx]

**Appendices**

Appendix 1: Search strategy MEDLINE (via PubMed)

Result: 1889 hits (1^st^ of April 2020)

1. health personnel[mh] OR geriatric nursing[mh]

2. healthcare[tiab] OR health-care[tiab] OR health care[tiab] OR social[tiab] OR hospital[tiab] OR clinic[tiab]

3. provider[tiab] OR providers[tiab] OR staff[tiab] OR personnel[tiab] OR specialist[tiab] OR specialists[tiab] OR professional[tiab] OR professionals[tiab] OR worker[tiab] OR workers[tiab] OR profession[tiab]

4. 2 AND 3

5. general practitioner[tiab] OR general practitioners[tiab] GP[tiab] OR nurse[tiab] OR nurses[tiab] OR nursing[tiab] OR physician[tiab] OR physicians[tiab] OR doctor[tiab] OR doctors[tiab] OR family practitioner[tiab] OR family practitioners[tiab] OR pharmacist[tiab] OR pharmacists[tiab] OR clinician[tiab] OR clinicians[tiab] OR paramedic*[tiab]

6. 4 OR 5

7. 1 OR 6

8. education[mh] OR teach-back communication[mh] OR health communication[mh] OR reminder system[mh]

9. educate[tiab] OR educates[tiab] OR educated[tiab] OR educating[tiab] OR education[tiab] OR educational[tiab] OR train[tiab] OR trains[tiab] OR trained[tiab] OR training[tiab] OR teach[tiab] OR teaches[tiab] OR taught[tiab] OR teaching[tiab] OR learn[tiab] OR learns[tiab] OR learned[tiab] OR learning[tiab] OR instruct[tiab] OR instructs[tiab] Or instructed[tiab] OR instructing[tiab] OR instruction[tiab] OR feedback[tiab] OR remind*[tiab] OR academic detailing[tiab] OR “peer comparison” [tiab] OR competition[tiab] OR outreach[tiab] OR workshop[tiab] OR seminar[tiab] OR quality improvement[tiab] OR quality assurance[tiab]

10. 8 OR 9

11. vaccines[mh] OR immunization[mh]

12. immunity[tiab] OR immunization[tiab] OR immunizations[tiab] OR immunizational[tiab] OR immunisation[tiab] OR immunisations[tiab] OR vaccin*[tiab] OR inoculat*[tiab]

13. 11 OR 12

14. adult[mh] NOT young Adult[mh]

15. health services for the aged[mh] 16. 14 OR 15

17. “older adult”[tiab] OR “older adults”[tiab] OR elderly[tiab] OR middle aged[tiab] OR “old age”[tiab] OR

senior[tiab] 18. 16 OR 17

19. matern*[ti] OR pregnan*[ti] OR parent*[ti] OR mother*[ti] OR father*[ti] OR pediatric*[ti] OR child[ti] OR childhood[ti] OR children[ti] OR toddler*[ti] OR infant*[ti] OR "young adult"[ti] OR "young adults"[ti] OR tuberculosis[ti] OR poliomyelitis[ti] OR mumps[ti] OR "cross-sectional"[ti]

20. qualitative research[majr]

21. cross-sectional studies[majr] 22. 19 OR 20 OR 21

23. 7 AND 10 AND 13 AND 18

24. 23 NOT 22

Searching query for copy and paste:

Search (((((((((((health personnel[mh] OR geriatric nursing[mh]))) OR (((((healthcare[tiab] OR health-care[tiab] OR health care[tiab] OR social[tiab] OR hospital[tiab] OR clinic[tiab])) AND (provider[tiab] OR providers[tiab] OR staff[tiab] OR personnel[tiab] OR specialist[tiab] OR specialists[tiab] OR professional[tiab] OR professionals[tiab] OR worker[tiab] OR workers[tiab] OR profession[tiab]))) OR (general practitioner[tiab] OR general practitioners[tiab] GP[tiab] OR nurse[tiab] OR nurses[tiab] OR nursing[tiab] OR physician[tiab] OR physicians[tiab] OR doctor[tiab] OR doctors[tiab] OR family practitioner[tiab] OR family practitioners[tiab] OR pharmacist[tiab] OR pharmacists[tiab] OR clinician[tiab] OR clinicians[tiab] OR paramedic*[tiab])))) AND (((education[mh] OR teach-back communication[mh] OR health communication[mh] OR reminder system[mh])) OR (educate[tiab] OR educates[tiab] OR educated[tiab] OR educating[tiab] OR education[tiab] OR educational[tiab] OR train[tiab] OR trains[tiab] OR trained[tiab] OR training[tiab] OR teach[tiab] OR teaches[tiab] OR taught[tiab] OR teaching[tiab] OR learn[tiab] OR learns[tiab] OR learned[tiab] OR learning[tiab] OR instruct[tiab] OR instructs[tiab] Or instructed[tiab] OR instructing[tiab] OR instruction[tiab] OR feedback[tiab] OR remind*[tiab] OR academic detailing[tiab] OR “peer comparison”[tiab] OR competition[tiab] OR outreach[tiab] OR workshop[tiab] OR seminar[tiab] OR quality improvement[tiab] OR quality assurance[tiab]))) AND (((vaccines[mh] OR immunization[mh])) OR (immunity[tiab] OR immunization[tiab] OR immunizations[tiab] OR immunizational[tiab] OR immunisation[tiab] OR immunisations[tiab] OR vaccin*[tiab] OR inoculat*[tiab]))) AND (((((adult[mh] NOT young Adult[mh])) OR health services for the aged[mh])) OR (“older adult”[tiab] OR “older adults”[tiab] OR elderly[tiab] OR middle aged[tiab] OR “old age” [tiab] OR senior[tiab])))) NOT (((matern*[ti] OR pregnan*[ti] OR parent*[ti] OR mother*[ti] OR father*[ti] OR pediatric*[Title] OR child[ti] OR childhood[ti] OR children[ti] OR toddler*[ti] OR infant*[ti] OR "young adult"[ti] OR "young adults"[ti] OR tuberculosis[ti] OR poliomyelitis[ti] OR mumps[ti] OR cross-sectional[ti] OR qualitative research[majr] OR cross-sectional studies[majr])))

Appendix 2: Search strategy Scopus

Result: 1614 hits (1^st^ of April 2020)

1. TITLE-ABS-KEY ( "general practitioner" OR gp OR nurs* OR physician OR doctor OR "family practitioner" OR pharmacist OR clinician OR paramedic* )

2. TITLE-ABS-KEY ( healthcare OR health-care OR "health care" OR social OR hospital OR clinic)

3. TITLE-ABS-KEY ( provider OR staff OR personnel OR specialist OR professional OR worker OR profession)

4. 2 AND 3

5. 1 OR 4

6. TITLE-ABS-KEY ( educate OR educates OR educated OR educating OR education OR educational OR train OR trains OR trained OR training OR teach OR teaches OR taught OR teaching OR learn OR learns OR learned OR learning OR instruct OR instructs OR instructed OR instructing)

7. TITLE-ABS-KEY ( instruction OR feedback OR remind* OR "academic detailing" OR "peer comparison" OR competition OR outreach OR workshop OR seminar OR "quality improvement" OR "quality assurance" )

8. 6 OR 7

9. TITLE-ABS-KEY ( immunity OR immunization OR immunizational OR immunisation OR vaccin* OR inoculat*)

10. TITLE-ABS-KEY ( elderly OR middle AND aged OR "old age" OR senior OR old AND adult)

11. TITLE ( matern* OR pregnan* OR parent* OR mother* OR father* OR pediatric* OR child* OR toddler OR "young adults" OR tuberculosis OR poliomyelitis OR mumps OR "cross-sectional")

12. 5 AND 8 AND 9 AND 10

13. 12 AND NOT 11

Searching query for copy and paste:

( ( ( TITLE-ABS-KEY ( "general practitioner" OR gp OR nurs* OR physician OR doctor OR "family practitioner" OR pharmacist OR clinician OR paramedic* ) ) OR ( ( TITLE-ABS-KEY ( healthcare OR health-care OR "health care" OR social OR hospital OR clinic ) ) AND ( TITLE-ABS-KEY ( provider OR staff OR personnel OR specialist OR professional OR worker OR profession ) ) ) ) AND ( ( TITLE-ABS-KEY ( educate OR educates OR educated OR educating OR education OR educational OR train OR trains OR trained OR training OR teach OR teaches OR taught OR teaching OR learn OR learns OR learned OR learning OR instruct OR instructs OR instructed OR instructing ) ) OR ( TITLE- ABS-KEY ( instruction OR feedback OR remind* OR "academic detailing" OR "peer comparison" OR competition OR outreach OR workshop OR seminar OR "quality improvement" OR "quality assurance" ) ) ) AND ( TITLE-ABS-KEY ( immunity OR immunization OR immunizational OR immunisation OR vaccin* OR inoculat* ) ) AND ( TITLE-ABS-KEY ( elderly OR middle AND aged OR "old age" OR senior OR old AND adult ) ) ) AND NOT ( TITLE ( matern* OR pregnan* OR parent* OR mother* OR father* OR pediatric* OR child* OR toddler OR "young adults" OR tuberculosis OR poliomyelitis OR mumps OR "cross-sectional" ) )

Appendix 3: Search strategy Cochrane library

Result: 143 hits (19^th^ of May 2020)

1. ( "general practitioner" OR gp OR nurs OR physician OR doctor OR "family practitioner" OR pharmacist OR clinician OR paramedic) OR ((healthcare OR health-care OR "health care" OR social OR hospital OR clinic) AND (provider OR staff OR personnel OR specialist OR professional OR worker OR profession))

2. educat OR train OR teach OR learn OR instruct OR feedback OR remind OR "academic detailling" OR "peer comparison" OR competition OR outreach OR workshop OR seminar OR "quality improvement" OR "quality assurance"

3. immunit OR vaccin OR inoculat

4. elderly OR senior OR old age OR older adult OR middle aged OR old AND adult

5. matern OR pregnan OR parent OR mother OR father OR pediatric OR child OR childhood OR toddler OR "young adults" OR tuberculosis OR poliomyelitis OR mumps OR cross-sectional

Searching query for copy and paste:

("general practitioner" OR gp OR nurs OR physician OR doctor OR "family practitioner" OR pharmacist OR clinician OR paramedic) OR ((healthcare OR health-care OR "health care" OR social OR hospital OR clinic) AND (provider OR staff OR personnel OR specialist OR professional OR worker OR profession)) in Title Abstract Keyword AND educat OR train OR teach OR learn OR instruct OR feedback OR remind OR "academic detailling" OR "peer comparison" OR competition OR outreach OR workshop OR seminar OR "quality improvement" OR "quality assurance" in Title Abstract Keyword AND immunit OR vaccin OR inoculat in Title Abstract Keyword AND elderly OR senior OR old age OR older adult OR middle aged OR old AND adult in Title Abstract Keyword NOT matern OR pregnan OR parent OR mother OR father OR pediatric OR child OR childhood OR toddler OR "young adults" OR tuberculosis OR poliomyelitis OR mumps OR cross-sectional in Record Title

- (Word variations have been searched)

Appendix 4: Search strategy grey literature

We searched the following grey literature sources:

- European Disease Control (ECDC)
- World health Organization (WHO)
- OpenGrey:

Searches OpenGrey http://www.opengrey.eu/

AND is the default boolean between terms.

- Vaccin* adult* 155 results

- Immuniz* adult* 12 results

- Immunis* adult* 21 results

- Vaccin* elder* 14 results

- Immuniz* elder* 1 result

- Immunis* elder* 4 results

Total = 207 results

- Grey Literature Report

Searches Grey Literature Report http://www.greylit.org/

AND is the default boolean between terms. Truncation automatically from six characters and up.

- Vaccin adults 26 results

- immunization adults 24 results

- immunisation adults 1 result

- Vaccin elderly 6 results

- Immuniz elderly 0 results

- Immunis elderly 0 results

Total = 57 results

Appendix 5: List of collected data items

| **Data domain** | **Data collected** |
| --- | --- |
| Study details | - First author, year - Title - Sponsorship Source - Conflict of interest - Country / geographical location - Healthcare setting - Comments - Research question / aim - Main conclusion - Author’s reasoning why the study was / was not successful |
| Author’s contact details | - Author’s name - Email - Address - Institution (from all authors) |
| Methods | - Study design - Mixed methods study? - Total study period - Type of addressed vaccine(s) - Pre-specified outcomes |
| Reference population of health care workers | - Inclusion and exclusion criteria - Group differences at baseline - Number of included HCWs / clinics - Mean age - Percentage of females |
| Reference population of target group (patients) | - Inclusion and exclusion criteria - Group differences at baseline - Number of included patients - Mean age - Percentage of females |
| Intervention | - Name of intervention(s) - Short description of intervention - Who provided it? - Where was it provided? - Drop-out and/or omission rate (missing data) - Description of intervention - Didactical methods used in the intervention - Educational content of the intervention - Reminding elements - Other |
| Outcomes (extracted separately for before and after data) | - Outcome name (i.e. influenza vaccination rates) - Outcome type(i.e. change in vaccination coverage) - Time-point - Patient population type (i.e. eligible patients) - Number of participants to whom data refers (denominator) - Scale/Range - Value (%, n, mean value, OR,RR, etc.) - Notes (i.e. statistical method and significance level, method of adjusting) |

Heterogeneity of denominators used for calculating vaccination rates

When estimating the proportion of vaccinated patients, studies may differ in their applied patient pool. Proportion measures are highly sensitive to the applied denominator, as at a constant number of performed vaccinations, the smaller the patient pool is, the higher the vaccination rate we get. Therefore, we registered the ‘patient population type’ into the data extraction table according to the following categories:

- All registered patients in the medical database
- Eligible patients (with indication, no contraindication and not vaccinated)
- Attending patients (not necessarily being eligible for vaccination)
- Eligible AND attending patients (eligible patients who visited the physicians)

As our narrative synthesis is based on comparing the absolute changes on the different intervention arms and does not aim for a cross-study comparison of absolute vaccination rates, we will not stratify our results according to the above mentioned categories. However, we registered the type of patient pool used in our data extraction table in case future researchers intend to perform further analyses.

Appendix 6: GRADE checklist nonrandomized studies

| 1. Failure to develop and apply appropriate eligibility criteria (inclusion of control population) | Under- or over-matching in case-control studies |
| --- | --- |
|  | Selection of exposed and unexposed in cohort studies from different populations |
| Reasoning/relevant part from the article | |
| 2. Flawed measurement of both exposure and outcome | Differences in measurement of exposure (e.g. recall bias in case-control studies) |
|  | Differential surveillance for outcome in exposed and unexposed in cohort studies |
| Reasoning/relevant part from the article | |
| 3. Failure to adequately control confounding | Failure of accurate measurement of all known prognostic factors |
|  | Failure to match for prognostic factors and/or adjustment in statistical analysis |
| Reasoning/relevant part from the article | |
| 4. Incomplete or inadequately short follow-up | Especially within prospective cohort studies, both groups should be followed for the same amount of time. |
| Reasoning/relevant part from the article | |
| 5. Overall judgment | |
| Reasoning/relevant part from the article | |

Agreements for assessment of the risk of bias in non-randomized studies:

- In case there is a ”failure to adequately control confounding” we will automatically assign high risk of bias for the given outcome. When making the overall judgement, provide a detailed description why you decide on low/high/some concerns, this enhances transparency.
- Incomplete or inadequately short follow-up: If the authors themselves state this as a limitation, and for example when the follow-up end before the flu season has ended (in case of the intervention targeting the uptake of the flu vaccine)

Appendix 7: Characteristics of the included studies (extended version)

|  | | | | | Nr. of HCWs | | Nr. of patients | |  | |
| --- | --- | --- | --- | --- | --- | --- | --- | --- | --- | --- |
|  | Healthcare setting | Patients targeted | Study type | Intervention type(s) | Intervention | Control | Intervention | Control | Vaccine type(s) | Main outcome(s) |
| Calkins, Katz [18] | Primary care | 65+ | Cluster RCT | Small-group consensus process | 17 Primary care providers (5 practices) | 19 Primary care physicians (4 practices) | 45 | 45 | Influenza | Vaccination rates |
| Chambers, Balaban [19] | Primary care | 65+ or risk groups | RCT | Reminder (digital) | Physicians (n=32) were stratified by level of training and randomized to one of three groups, | See under ‘Intervention’ | -Always reminded: 271  -Sometimes reminded: 146 | -Never reminded: 218 | Influenza | Vaccination rates |
| Chan, MacLehose and Houck [42] | Solo and group practices of physiatrists (outpatient) | Patients with a chronic medical condition | RCT with cross-over design | Reminder (paper) | -Solo practices 1997: 23  -Group practices 1997: 28  -Solo practices 1998: 20  -Group practices 1998: 32 | -Solo practices 1997: 21  -Group practices 1997: 33  -Solo practices 1998: 20  -Group practices 1998: 27 | -Solo practices 1997: 1486  -Group practices 1997: 1341  -Solo practices 1998: 561  -Group practices 1998: 868 | -Solo practices 1997: 596  -Group practices 1997: 877  -Solo practices 1998: 1310  -Group practices 1998: 1286 | Influenza | Vaccination rates |
| Changolkar, Rewley [58] | Primary care | Mean age (SD): 58.7 (16.3) years | Prospective cohort study | Reminder | Low clin. workload: 12 physicians  High clin. workload: 18 physicians | Low clin. workload: 3 physicians  High clin. workload: 23 physicians | Low clin. workload: before:1974 (pre-intervention), 1799 (post)  High clin. workload: 6397 (pre-intervention), 5342 (post) | Low clin. workload:558 (pre-intervention), 516 (post)  High clin. workload:14275 (pre-intervention), 14549 (post) | Influenza | Vaccination rates |
| Cohen, Littenberg [20] | General medical clinic (outpatient) | 65+ | Cluster RCT | Education + reminder (checklist, paper) | 22 Physicians (2 firms) | 1 Firm (no info on number of physicians) | Eligible patients  -Pneumovax: 547  -Influenza: 581 | Eligible patients  -Pneumovax: 291  -Influenza: 291 | Influenza  Pneumococcal | -Vaccination rates  -Knowledge |
| Cowan, Heckerling and Parker [21] | General medical clinic (outpatient) | >65 influenza and pneumococcal.  Tdap every ten years | Cluster RCT | Reminder (recommend-dations, paper) | 16 Residents | 13 Residents | 62 | 45 | Influenza  Pneumococcal  Tdap | -Vaccination coverage  -Knowledge  -Attitude |
| Crouse, Nichol [22] | Hospital (inpatient) | High-risk patients | Prospective cohort study | Comparison of:  -standing  orders  -physician education  -Physician  reminder  (paper) | 6 hospitals in total; 2 hospitals per intervention type | n/a | -Standing order group: 609  -Physician reminder group: 1925  Physician education group: 11800 | n/a | Influenza | -Proportion of offered vaccination  -Vaccination rates |
| Desai, Lu [59] | Hospital Arthritis center + 4 of its satellite ambulatory practices (outpatient) | -65 years and older  - <64 when in immunocompromising condition/ immunosuppressive therapy | Prospective cohort study | Reminder (paper) | 14 rheumatologists | 21 rheumatologists | 3267 | 450 | Pneumococcal | Vaccination rates |
| Dexter, Perkins [43] | Hospital (inpatient) | NI | RCT | Reminder (digital) | -96 (47.5%) physicians intervention group only  -28 (13.9%) physicians assigned alternately to both intervention and control group | - 78 (38.6%) physicians control group only | 4995 hospitalizations | 5070 hospitalizations | Influenza  Pneumococcal | Proportion of offered vaccination |
| Dexter, Perkins [44] | Hospital: (inpatient) | - 65 years and older or risk group (relevant chronic disease) | Cluster RCT | Comparison of:  - Standing  orders  (digital)  -Physician Reminders (digital) | 4 teams assigned to physician reminder group | 4 teams assigned to standing order group | 691 Patients eligible for vaccination | 623 Patients eligible for vaccination | Influenza  Pneumococcal | Vaccination rates |
| Dubey, Mathew [45] | primary care | Patients aged 21 years and older | Cluster RCT | Reminder (paper) | 20 primary care physicians | 18 primary care physicians | 248 | 261 | Tetanus  Rubella | Proportion of offered vaccination |
| Flanagan, Doebbeling [23] | Primary Care | -Influenza: hospital employee or older than 64.5 years of age  -Pneumococcal: recommended if age was greater than 64 years (flagged "consider" if more than 63.5 years) and more than 10 years since last  received (flagged "consider" if more than 7 years  since last received).  Td: recommended if no history of Tetanus vaccine in over 9 years and 6 months. | RCT with cross-over design | Reminder (digital) | 70 physicians | 47 physicians | NI | NI | Influenza  Pneumococcal  Tetanus  Hepatitis  Measles | Proportion of vaccines ordered |
| Hohmann, Hastings [60] | community pharmacies | Unclear | Cluster RCT | Comparison of:  -Multicomponent intervention  -Online webinar | 30 community pharmacies | 32 control community pharmacies | NI | NI | Pneumococcal  Herpes zoster | -Vaccination coverage  -Organiza-tion level |
| Hutchison [68] | Group family practice | 65 years and older | Controlled interrupted time series | Reminder (paper) | NI | NI | Eligible patients: 593 | Eligible patients: 618 | Influenza | -Vaccination rates  -Patient refusal rate |
| Jans, Schellevis [46] | Primary care | 16-70 years of age and having received a diagnosis of asthma or COPD | Retrospective cohort study | Multicomponent intervention: quality system | 14 practices (total 16 physicians) | 5 practices (totalling 5 physicians) | 455 | 152 | Influenza | Vaccination rates |
| Karuza, Calkins [24] | Primary care physician group practices | 65 years and older | Cluster RCT | Small-group consensus process | 23 primary care physicians | 28 primary care physicians | Mean number of charts reviewed per physician: 30 | Mean number of charts reviewed per physician: 29 | Influenza | -Vaccination rates  -Attitude  -Knowledge |
| Kerse, Flicker [25] | General practices | 65 years and older | RCT | Multicomponent intervention:  -Clinical practice audit with feedback  -Educational detailing  -Card based prompt system  -Seminar or home based learning  -Resource directory | 21 General practitioners | 21 General practitioners | 121 | 112 | Influenza | -Vaccination rates |
| Kiefe, Allison [47] | Family medicine, internal medicine, and endocrinology (outpatient setting) | 65 years and older with diabetes mellitus | Cluster RCT | Comparison of:  -multi-component intervention with performance feedback  -Same multicomponent intervention + achievable benchmark feedback | 35 Physicians | 35 Physicians | 965 | 966 | Influenza | -Vaccination rates |
| Kim, Kristopaitis [26] | Group-model health maintenance organization | Patients aged 65 to 75 years | RCT | Comparison of:  -Education only  -Multicomponent intervention | Multicomponent intervention:21 primary care physicians | Education only: 20 primary care physicians | Multicomponent intervention: 905 | Education only: 905 | Influenza  Pneumococcal  Tetanus | -Proportion of vaccines offered  -Vaccination rates |
| Klein and Adachi [27] | Hospital (inpatient) | -Patients of 65 years or older  -Patients with high-risk conditions | RCT | Reminder (paper) | NI | NI | 100 | 100 | Influenza | Vaccination rates |
| Korn, Schlossberg and Rich [28] | Outpatient Clinic | 65 years and older | Retrospective cohort study | Multicomponent intervention | 15 Residents | 13 Residents | -SPR site post-intervention: 199  -VA site: 149 | -SPR site pre-intervention: 202  -VA site: 151 | Influenza  Pneumococcal | Proportion of vaccines ordered |
| Lemelin, Hogg and Baskerville [48] | Community primary care practices (Health Service Organizations (HSOs)) | 65 years and older | Cluster RCT | Multicomponent intervention | 22 Practices | 23 Practices | NI | NI | Influenza | Vaccination rates |
| Loo, Davis [61] | General Medicine and Primary Care Division | 65 years and older | Prospective cohort study | Comparison of  -Reminder (digital)  -Reminder (digital) + panel manager  -Control | -Reminder: 17 primary care physicians  -Reminder + panel manager: 17 primary care physicians | Control: 20 primary care physicians | -Reminder: 1336  -Reminder + panel manager: 1394 | Control: 1930 | Influenza  Pneumococcal | Vaccination rates |
| Loskutova, Smail [62] | Primary care | Influenza: 18 years and older  Pneumococcal:  -65 years and older  -19-64 years when at least one risk factor  Herpes zoster: 60 years and older | Prospective cohort study | Comparison of:  -Multicomponent intervention  - Reminders + Clinical decision support system (algorithms for provider reminders and standing orders) | 23 Primary care physicians | 20 Primary care physicians | -Influenza year 1 (before): 20,952  -Influenza year 2 (after): 24,506  -Pneumococcal year 1 (before): 18,244  -Pneumococcal year 2 (after): 27,415  -Zoster year 2 (before): 27,415  -Zoster year 3 (after): 30,844 | -Influenza year 1 (before): 15,076  -Influenza year 2 (after): 17,256  -Pneumococcal year 1 (before): 12,577  -Pneumococcal year 2 (after): 21,465  -Zoster year 2 (before): 21,465  -Zoster year 3 (after): 24,906 | Influenza  Pneumococcal  Herpes zoster | Vaccination rates |
| MacIntyre, Kainer and Brown [49] | Hospital (inpatient) & community  General practitioners | 65 years and older | RCT | Comparison of:  -Hospital reminder (paper + verbal)  -GP reminder (letter) | NI | NI | Hospital reminder: 70  GP reminder: 61 | n/a | Influenza  Pneumococcal | Vaccination rates |
| McDonald, Hui [29] | General medicine clinic | Influenza: according to U.S. Public health service criteria  Pneumococcal: Over 65 years, otherwise according to U.S. Public health service criteria | Cluster RCT | Reminder (paper) | 61 Residents | 54 Residents | NI | NI | Influenza  Pneumococcal | -Intentions among HCWs  -Proportion of vaccines offered |
| McDonald, Hui and Tierney [30] | General medicine clinic | Older than 65 years or risk group (chronic lung disease, asthma, diabetes mellitus, congestive heart failure, severe renal/hepatic failure) | RCT | Reminder (digital) | NI | NI | -Intervention group 1978-1979: 1328  -Intervention group 1979-1980: 1489  -Intervention group 1980-1981: 1316 | -Control group 1978-1979: 1290  -Control group 1979-1980: 1412  -Control group 1980-1981: 1236 | Influenza | -Vaccination coverage  -Disease incidence |
| McGreevy, McGowan [63] | Resident physician continuity clinic | 65 Years and older | Prospective cohort study | Multicomponent | 2 Attending physicians and 8 residents | Not explicitly stated | ≥65 years: 95  ≤65 years: 255 | ≥65 years: 103  ≤65 years: 242 | Pneumococcal | Vaccination rates |
| Ngamruengphong, Horsley-Silva [64] | Academic institutions | Patients with diabetes mellitus | RCT | Comparison of:  -Standard education + extra 30-minute lecture + pocket card + monthly e-mail reminders with the lecture content  -Standard education | 20 Primary care residents | 19 Primary care residents | 29 | 17 | HBV | Knowledge |
| Nowalk, Nutini [65] | primary care practices | Influenza: all adults  Pneumococcal: 65 years and older or high-risk patients aged 18-64. | Prospective cohort study | Multicomponent: Toolkit | 10 providers and 7 clinical assistants | 2 providers and 3 clinical assistants | 7794 | 2199 | Influenza  Pneumococcal | Vaccination coverage |
| Ornstein, Garr [31] | Family Medicine Center | 18 years or older | RCT | Comparison of:  -Educational + administrative interventions  -abovementioned interventions + reminders (paper) | -Physician reminders: 14  -Patient reminders: 12  -Physician and patient reminders: 13 | Control: 10 | -Physician reminders: 1988  -Patient reminders: 1925  -Physician and patient reminders: 1908 | Control: 1576 | Tetanus | Vaccination coverage |
| Overhage, Tierney and McDonald [32] | general medicine (inpatient) | Older than 65 years or in risk group (Reactive airway disease,  congestive heart failure, diabetes, splenectomy, sickle  cell disease) | RCT | Reminder (digital and paper) | 12 teams of physicians and medical students | 12 teams of physicians and medical students | 821 | 801 | Pneumococcal | Compliance with preventive care guidelines |
| Quinley and Shih [50] | Primary care practices | 65 years and older | Cluster RCT | Comparison of:  -Mailer only  -Mailer + telephone follow-up | -African-American practices: 118  -High volume practices: 582 | -African-American practices: 100  -High volume practices: 150 | NI | NI | Pneumococcal | Vaccination rates |
| Rosser, McDowell and Newell [34] | Civic Hospital Family Medicine Centre | Influenza: Older than 65 years  Tetanus: over 18 years of age | RCT | Comparison of:  -Physician reminder (paper)  -Letter reminder to patients  -Telephone reminder to patients | -Six teams consisting of a staff physician, nurse, and three to five residents. No information was provided on the distribution of these HCPs per study group. | NI | -Physician reminder: 1471  -Letter reminder: 1541  -Telephone reminder | -Nonrandomized control: 2619  -Randomized control: 1403 | Influenza  Tetanus | Vaccination rates |
| Rosser, Hutchison [33] | Civic Hospital Family Medicine Centre (outpatient) | patients 20 years of age or more | Cluster RCT | Comparison of:  -Physician reminder (paper)  -Letter reminder to patients  -Telephone reminder to patients | 4 Teaching medical practices participated, but no information was provided on the distribution of these HCPs per study group. | NI | -Physician reminder: 1399  -Letter reminder: 1471  -Telephone reminder: 1390 | Control: 1329 | Tetanus | Vaccination rates |
| Schreiner, Petrusa [35] | Resident outpatient clinics | -65 years and older or when having a chronic disease | Prospective cohort study | -Five months with reminders (paper) followed by six months follow-up without reminders | 20 Residents | 22 Residents | 1260 | 504 | Pneumococcal | Proportion of vaccines offered |
| Shevlin, Summers-Bean [51] | Hospital (inpatient) | -65 years and older or when in risk group (diabetes, alcohol abuse, lung/heart disease, HIV, chronic renal failure) | Prospective cohort study | -Reminders (paper) + in-service education + feedback | 2 Floors | 2 Floors | 296 | 238 | Pneumococcal | Vaccination rates |
| Shultz, Malouin [66] | -Family medicine clinics  -Internal medicine clinics (control group) | 11- to 64-year-old patients | Prospective cohort study | Intervention consisting of:  -Reminder (digital)  -Pay-for-performance  -Monthly status reports | 5 Family medicine clinics | 4 Primary care clinics | 39882 | 28032 | Tdap | Vaccination rates |
| Siriwardena [52] | Primary care | -65 years and older or when in risk group (coronary heart  disease, diabetes, or splenectomy) | Cluster RCT | Comparison of:  -Educational outreach based on principles of academic detailing + audit feedback and written guidance  -Audit feedback and written guidance | 15 | 15 | Influenza vaccination 65+: 13633 | Influenza vaccination 65+: 13947 | Influenza | Vaccination rates |
| Solberg, Kottke [53] | Private primary care clinic | Older than 64 years | Cluster RCT | - Leadership involvement, training, network and consultation | 22 Clinics | 22 Clinics | 3379 | 3451 | Pneumococcal | Vaccination rates |
| Stevenson, McMahon [54] | long-term care facilities (LTCFs) | All residents | Prospective cohort study | Comparison of 4 slightly different multicomponent interventions | Alaska: 26 facilities  Idaho: 23 facilities  Montana: 78 facilities  Wyoming: 6 facilities | n/a | Alaska: 1099  Idaho: 1274  Montana: 5671  Wyoming: 882 | n/a | Pneumococcal | Vaccination rates |
| Tang, LaRosa [36] | Internal medicine clinic | patients 65  years of age and older | Prospective cohort study | Reminder (digital) | 13 | 10 | NI | NI | Influenza | Behaviour among HCWs |
| Tape and Campbell [37] | General internal medicine clinic | Influenza: 65 years and older or when in risk group (diabetes, chronic respiratory or heart disease)  Pneumococcal: 65 years and older or when in risk group (immunocompromised, diabetes, chronic respiratory or heart disease)  Tetanus: all patients | Prospective cohort study | Comparison of:  -Education + flow sheet  -Education + reminder (six months on paper, then six months displayed on terminals) | 45 residents in total and 4 attending physicians. No information on the numbers assigned to the control/interven-tion arm |  | Tetanus: 937  Influenza: 212  Pneumococcal: 310 | Tetanus: 870  Influenza: 172  Pneumococcal: 274 | Influenza  Pneumococcal  Tetanus | Behaviour among HCWs |
| Tierney, Hui and McDonald [38] | General medicine clinic | Unclear | Cluster RCT | Various combinations feedback and reminders (paper) | -Group A feedback and A reminders: 33  - Group A feedback and B reminders: 31  -Group B feedback and A reminders: 36 | Group B feedback and B reminders: 35 | -Group A feedback and A reminders: 1487  - Group A feedback and B reminders: 1451  -Group B feedback and A reminders: 1606 | Group B feedback and B reminders: 1501 | Pneumococcal | Behaviour among HCWs |
| Trick, Das [55] | Hospital (inpatient) | All patients (median age: 52 years) | Prospective cohort study | Comparison of:  -standing-orders policy  -augmentation of the standing-orders  policy with electronic opt-out orders for physicians  - augmentation of the standing-orders  policy with electronic  reminders to nurses | NI | NI | -Nursing reminder: 69  -Opt-out: 66 | Control:69 | Influenza | Vaccination rates |
| Turner, Waivers and O'Brien [40] | Outpatient center | Both vaccines: older than 65 or in risk group | RCT | Comparison of:  -Physician reminder (paper)  -Physician reminder (paper) + reminder card carried by patients | 12 Residents | 12 Residents | 117 | 246 | Influenza  Pneumococcal | Vaccination coverage |
| Turner, Peden and O'Brien [39] | Private practice offices | 65 years or older or suffering from a chronic disease | RCT | Comparison of:  -Physician reminder (paper)  - Reminder card carried by patients | -Computer-generated reminder: 15 physicians  -Patient-carried reminder: 22 physicians | n/a | -Computer-generated reminder: 300  -Patient-carried reminder: 440 | n/a | Influenza | Vaccination coverage |
| van Essen, Kuyvenhoven and de Melker [41] | Primary care | All patients | Prospective cohort study | Guideline for influenza vaccination | 64 practices (84 GPs) | 74 practices (88 GPs) | about 250,000 | about 300,000 | Influenza | -Vaccination coverage  -Organiza-tion level |
| Warner and Seleznick [56] | Hospital-based ambulatory clinic | patients 65 years of age or older | Prospective cohort study | Comparison of:  -Education + reminder (paper)  -Education only | 1 Clinic | 1 Clinic | NI | NI | Pneumococcal | -Vaccination rates  -Organiza-tion level |
| Winston, Lindley and Wortley [57] | Hospital (inpatient) | patients aged 50 years and older | Retrospective cohort study | Comparison of:  -A. Nurse-administered standing orders protocol + Training and information: Small group meetings on individual units  -B. Physician reminder program + Training and information: E-mail, newsletters, and division meetings  -C+D Nurse-administered standing orders protocol + Training and information: Nurse managers trained during regular staff meetings | A: 93 (50-64y, 2002)  90 (50-64y, 2003)  93 (65+y, 2002)  87 (65+y, 2003)  B: 39 (50-64y, 2002)  49 (50-64y, 2003), 98 (65+y, 2002)  114 (65+y, 2003)  C: 109 (50-64y, 2002), 95 (50-64y, 2003), 110 (65+y, 2002), 96 (65+y, 2003)  D 112 (50-64y, 2002)  91 (50-64y, 2003)  102 (65+y, 2002)  98 (65+y, 2003) | n/a | 4 Hospitals (A-D) | n/a | Influenza  Pneumococcal | Vaccination rates |
| Yi, Zhou [67] | No healthcare setting but two streets in Ningbo city | 60 years and older + chronic disease | Prospective cohort study | Education | NI | NI | 7013 | 5500 | Influenza | Vaccination coverage |

*NI = ‘No information available’, n/a = not applicable*
